# A HAD family phosphatase CSP-6 regulates the circadian output pathway in *Neurospora crassa*

**DOI:** 10.1371/journal.pgen.1007192

**Published:** 2018-01-19

**Authors:** Xiaoying Zhou, Bin Wang, Jillian M. Emerson, Carol S. Ringelberg, Scott A. Gerber, Jennifer J. Loros, Jay C. Dunlap

**Affiliations:** 1 Department of Molecular and Systems Biology, Geisel School of Medicine, Dartmouth, Hanover, New Hampshire, United States of America; 2 Biochemistry and Cell Biology, Geisel School of Medicine, Dartmouth, Hanover, New Hampshire, United States of America; 3 Norris Cotton Cancer Center, Geisel School of Medicine at Dartmouth, Hanover, New Hampshire, United States of America; Rutgers, The State University of New Jersey, UNITED STATES

## Abstract

Circadian clocks are ubiquitous in eukaryotic organisms where they are used to anticipate regularly occurring diurnal and seasonal environmental changes. Nevertheless, little is known regarding pathways connecting the core clock to its output pathways. Here, we report that the HAD family phosphatase CSP-6 is required for overt circadian clock output but not for the core oscillation. The loss of function Δ*csp-6* deletion mutant is overtly arrhythmic on race tubes under free running conditions; however, reporter assays confirm that the FREQUENCY-WHITE COLLAR COMPLEX core circadian oscillator is functional, indicating a discrete block between oscillator and output. CSP-6 physically interacts with WHI-2, Δ*whi*-2 mutant phenotypes resemble Δ*csp-6*, and the CSP-6/WHI-2 complex physically interacts with WC-1, all suggesting that WC-1 is a direct target for CSP-6/WHI-2-mediated dephosphorylation and consistent with observed WC-1 hyperphosphorylation in Δ*csp*-6. To identify the source of the block to output, known clock-controlled transcription factors were screened for rhythmicity in Δ*csp*-6, identifying loss of circadian control of ADV-1, a direct target of WC-1, as responsible for the loss of overt rhythmicity. The CSP-6/WHI-2 complex thus participates in the clock output pathway by regulating WC-1 phosphorylation to promote proper transcriptional/translational activation of *adv*-1/ADV-1; these data establish an unexpected essential role for post-translational modification parallel to circadian transcriptional regulation in the early steps of circadian output.

## Author summary

Though molecules and components in the core circadian oscillator are well studied in *Neurospora*, the mechanisms through which output pathways are coupled with core components are less well understood. In this study we investigated a HAD phosphatase, CSP-6; loss-of-function Δ*csp*-6 strains are overtly arrhythmic but have a functional core circadian oscillation. CSP-6 in association with WHI-2 dephosphorylates the core clock component WC-1 to regulate light-responses and development. To dissect the functions of CSP-6 in core clock and output, we screened known WC-1 targets and found that loss of CSP-6 causes misregulation of transcriptional/translational activation of ADV-1, a key regulator of output. Thus, loss of CSP-6-mediated dephosphorylation of WC-1 leads to loss of ADV-1 activation and is responsible for the complete loss of overt developmental rhythmicity in Δ*csp*-6.

## Introduction

Circadian clocks are endogenous timekeepers that control a wide variety of daily biochemical, physiological, molecular and behavioral rhythms in mammals, plants, insects, fungi and cyanobacteria. The circadian system consists of three essential parts, input, a central oscillator and output [1–4]. In fungi and animals, the backbone of the oscillator mechanism is a transcriptional and translational autoregulatory feedback loop driven by positive and negative elements. The positive element, a heterodimeric transcription factor in which the proteins interact via PAS domains, drives expression of the negative element, a complex of proteins that physically interacts with the positive element to reduce its activity. In the case of *Neurospora crassa*, the positive elements are the PAS domain containing transcription factors White Collar-1 (WC-1) and White Collar-2 (WC-2) that form the White Collar Complex (WCC). WCC in turn activates transcription of the negative element gene *frequency (frq*); FRQ nucleates formation of a complex including FRQ Interacting RNA Helicase (FRH) and Casein Kinase 1 (the FRQ/FRH Complex or FFC) that feeds back to physically interact with WCC and suppress *frq* transcription [5–7]. FRQ is progressively phosphorylated over time, modifications that provide the long time constant for the cycle and that ultimately reduce the affinity of the FFC for the WCC, releasing it to initiate the next cycle of transcription. Eventually hyperphosphorylated FRQ is turned over via a ubiquitin-mediated pathway, but in a normal circadian cycle the kinetics of this turnover is not believed to influence the period length of the clock [8,9].

Both WC-1 and WC-2 are phosphorylated *in vivo* under circadian conditions and become hyperphosphorylated after a short light exposures [10,11]. In the current model of the circadian feedback loop, the FRQ-FRH complex (FCC) closes the loop by inhibiting WCC activity *via* the promotion of phosphorylation of WCC, primarily through kinases CK-1a and CKII [12–14]. The importance of WCC phosphorylation for circadian oscillation has been argued based on short period, low amplitude, phase shifted and arrhythmic phenotypes resulting from mutations of phosphorylation sites on WCC [15–17]. In the current model, hyperphosphorylated WCC is believed to be inactive but stable whereas hypophosphorylation WCC is active and supports transcriptional activation of *frq* and other genes [12–14]. PP2A (protein phosphatase 2A) is believed to dephosphorylate WC-1 *in vivo* and this is correlated with an increase *frq* RNA levels [2,18].

In addition to its clock functions, WC-1 and WC-2 (WCC) comprise the blue light photoreceptor that initiates the organism’s principal photoresponse. Upon illumination the WCC undergoes a rapid conformation change, binding to light-responsive elements (LREs) via WC-2 and functioning as a TF to bind to and regulate the expression of hundreds of light-responsive genes [10,19–22]. Similar to WCC functioning in the dark, hyperphosphorylated WC-1 is believed to be transcriptionally inactive and hypophosphorylated WC-1 transcriptionally active. Consistent with this are reports that hyperphosphorylated WC-1 binds less strongly to target promoters while dephosphorylation of WC-1 increases promoter binding [23,24]. VVD (VIVID), a small PAS/LOV protein and another blue light receptor, acts as a repressor of the light response through its physical interaction with the WCC [11], and recent studies have shown the photocycle length of VVD plays a dominant role in determining the utility of the photoreceptor [25]. Though VVD is not required for clock rhythmicity, it modulates various WCC-mediated circadian clock properties such as gating of light input of clock and phasing light resetting responses. Loss of function *vvd* mutants exhibit a 4-hour delay of clock-controlled conidiation [26,27].

Time of day information generated by the circadian clock is transduced to clock control genes (*ccgs*) whose time-of-day specific expression yields products, the output pathway, that generate overt rhythmicity in the cell [28,29]. The best-characterized and most easily monitored output of the FFC/WCC Oscillator is the conidiation rhythm [30,31]. Though enormous advances have been seen in understanding core oscillators of *Neurospora* in the past two decades, how circadian oscillators signal through output pathways to control rhythmic activity of those *ccgs* remains only partially understood at molecular level [9,30]. Most recently, clock-controlled genes showing consistent rhythms and comprising as much as 40% of the genome have been identified in *N*. *cras*s*a* by RNA-seq [32]. While these will provide a great resource for studying rhythmic behavior in the future they have afforded limited insight into the connection between the core clock and individual outputs.

Here we characterize functions of a HAD-domain family phosphatase protein, CSP-6, revealing its essential role in regulating circadian output pathways including conidiation rhythms and phase resetting. CSP-6 physically interacts with a partner, WHI-2, and this phosphatase complex, which interacts with WC-1, is important for maintaining WC-1 protein and phosphorylation levels. Loss of function of *csp-6* results in constitutively hyperphosphorylated WC-1 but does not ablate core oscillator function, although the clock shows a 3.5-h phase delay due to reduced amounts and hyperphosphorylation of WC-1. This indicates that the regulation of genes and proteins acting downstream of WC-1 to control circadian developmental processes has been disrupted in Δ*csp*-6, and our results are consistent with a model in which ADV-1, a direct target of WC-1, plays this direct role in regulating overt clock output [33]. Prior to this finding the early steps of circadian output have been viewed primarily as a transcriptional network of activators and repressors. CSP-6 demonstrates an essential role for post-translational modifications in the early steps of output.

## Results

### CSP-6 is required for overt rhythmicity but retains a functional clock

**Fig 1:**
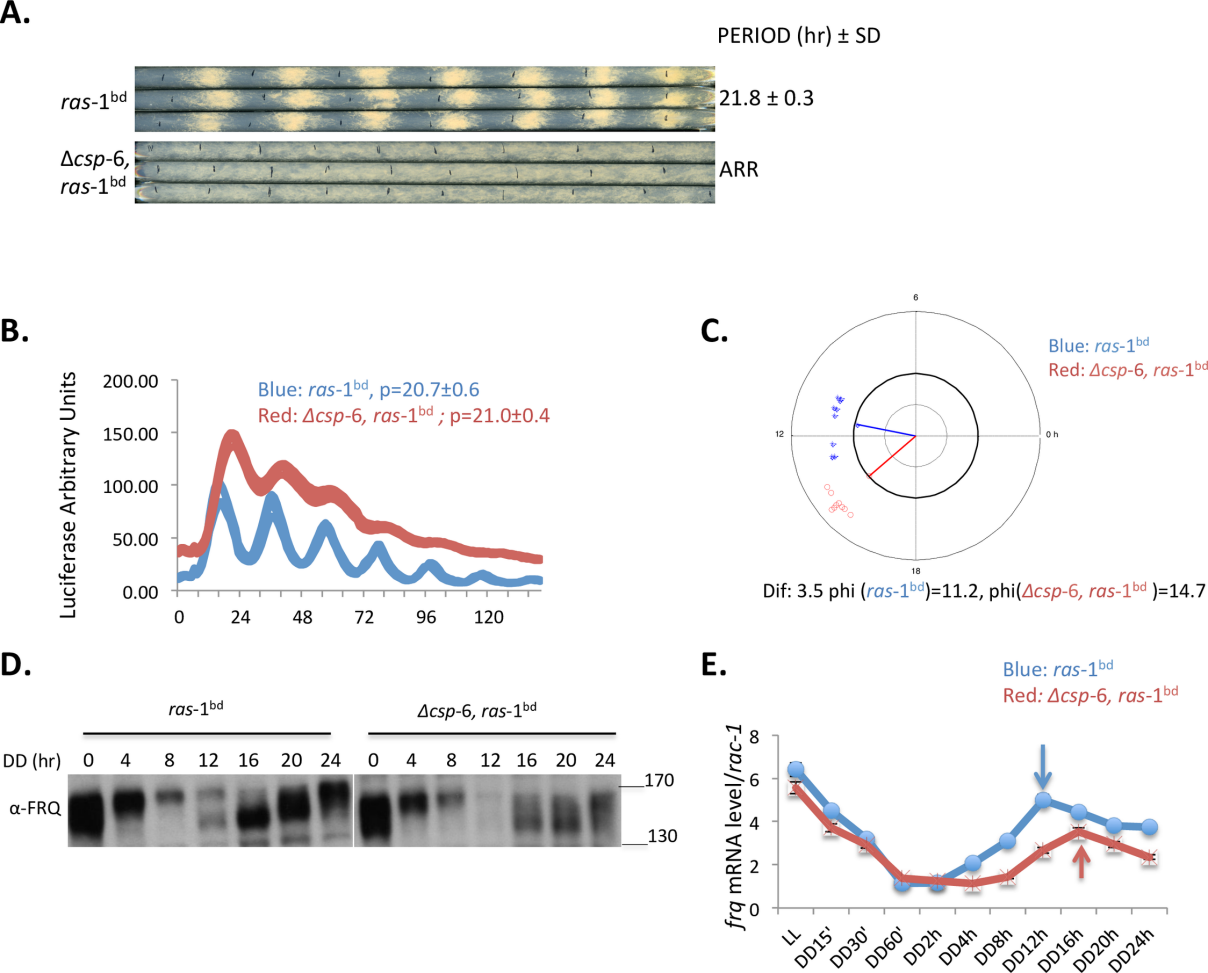
Loss of *csp-6* abolishes conidiation rhythms and results in a phase delay in *frq* transcriptional and FRQ translational rhythms. A: Race tube assays of WT *ras*-1^bd^ and Δ*csp-*6, *ras*-1^bd^ strains. Period is reported in hours ± one standard deviation, ARR is arrhythmic. B: Analyses of luciferase activity rhythms of *frq C-box-luc* in WT (blue) and Δ*csp-*6 (red). Strains were grown on race tube medium in a 96 plate under constant light for 48 hrs and then transferred to constant dark. The luciferase signals were captured every hour for 6 days. Three replicates for each strain are shown and ‘p’ stands for period. C: Determination of phase delay in Δ*csp-*6. Primary time series data from the experiments shown in panel B were analyzed using a Matlab-based program [60]; Dif. corresponds to the phase difference between *ras*-1^bd^ and Δ*csp-*6, *ras-*1^bd^, and phi reports the average phase of the rhythm in each genetic background. D: FRQ protein remains rhythmic in the Δ*csp-6* mutant. Shown is a Western blot of FRQ protein in *ras*-1^bd^ and Δ*csp-*6, *ras*-1^bd^, the hours in constant darkness are shown above the blots. Protein maker(s) were used as with the molecular weight (KDa) as indicated. E: *frq* mRNA accumulates with a circadian rhythm in the Δ*csp-6* mutant as it does in wild type. *frq* mRNA expression was assayed by qRT-PCR and normalized to *rac-*1 in *ras*-1^bd^ and *ras*-1^bd^,Δ*csp-*6; *frq* mRNA peaks at DD16 in Δ*csp-6* instead of DD12 in WT.

The *csp*-6 gene (NCU08380) encodes a member of the haloacid dehalogenase (HAD) superfamily containing a conserved dullard-like phosphatase domain, (S1A Fig, HAD domain 222–388 aa). It was first reported in a screen of putative phosphatases as having a conidial separation phenotype [34] and was later reported (under the name *psr*-1) to be more broadly involved in female sexual development, cell-cell-fusion and autophagy [35]. Because the conidial separation defect was similar to that seen in mutants of *csp*-1 and *csp*-2, two transcription factors important for conidiation and the circadian clock, we asked whether Δ*csp-*6 has similar phenotypes. We crossed the Δ*csp*-6 deletion mutant with the *ras*-1^bd^ mutant and performed race tube assays (Fig 1A) revealing that, unlike the *ras-*1^bd^ strain, Δ*csp*-6, *ras*-1^bd^ show reduced hyphal growth and arrhythmic conidiation. Use of *frq-luc* to report clock core oscillator function [36] in this background (Δ*csp*-6, *ras*-1^bd^, *csr*::*c-box-luc)*, however, revealed rhythmic *frq* expression with a wild type period length around 22 h, albeit having a somewhat reduced amplitude and a 3.5-hour phase delay (Fig 1B and 1C). While luciferase reporter data as seen in Fig 1B is an excellent indicator of rhythmicity because of the density of time points, the amount of bioluminescence produced is a function of growth and development as well as the clock; Δ*csp*-6-induced changes in growth characteristics could thus influence bioluminescent output, so we also examined *frq* and FRQ biochemically to more directly view the effect of Δ*csp*-6 on the oscillator components. Analysis of mRNA by RT-qPCR and protein by Western blotting confirmed core clock rhythmicity, showing that FRQ oscillated with a small loss of amplitude in Δ*csp*-6 mutants and displayed a delay of around 4 hours (Fig 1D), and *frq* mRNA levels rhythmic but again phase delayed about 4 hours (Fig 1E).

We also followed FRQ and *frq* levels from DD24h to DD48h during the second day after moving from LL to constant DD. The results showed FRQ protein levels were reduced but we still can observe rhythmicity in FRQ amounts along with its phosphorylation (S1B Fig), consistent with c-*box-luc* luciferase traces in Δ*csp*-6. The *frq* mRNA level was reduced in Δ*csp*-6 but the amount still oscillated with a peak time of DD36h, 4 hours delay compared to wild type (S1C Fig). These data indicated that CSP-6 plays a role in maintaining robust *frq/*FRQ expression but is not required for the clock core oscillation. So we concluded that the arrhythmic conidiation on race tubes in the Δ*csp-*6 mutant was caused by disruption of a circadian output pathway.

The *csp-*6 promoter shows very weak circadian regulation when assayed by luciferase fusion (S1D Fig), but *csp*-6 is not a typical clock-controlled gene involved in circadian output. The weak transcriptional regulation detected by luciferase is also seen in a translational fusion reporter of CSP-6 but not confirmed by Western analysis (S1E and S1F Fig), so its action appears more likely to facilitate output rather than to drive it. *Saccharomyces* contains both an ortholog and a paralog of *csp*-6, *psr*1 and *psr*2 respectively, both of which arose together in screens and share similar mutant phenotypes, interactors, and functions (http://www.yeastgenome.org/locus/S000004009/overview); in yeast these phosphatases function to regulate the stress response [37]. Likewise, *Neurospora* has a *csp-*6 paralog (NCU08948 provisionally named *pph-*11 [34]) that contains the same conserved HAD phosphatase domain as *csp*-6 (S2A Fig, underlined sequences). To investigate whether *csp*-6 and its paralog are both involved in circadian clock output, conidiation rhythms were followed on race tubes in ΔNCU08948 (*psr*-2, *ras-*1^bd^) (S2B Fig). The ΔNCU08948, *ras*-1^bd^ mutant showed normal overt rhythmicity although it grew a bit slower compared to the wild type. Assay of the core circadian oscillator via a *frq-luc* reporter confirmed a robust circadian rhythm (S2C Fig) indicating that only *csp-*6 but not its paralog is involved in circadian function in *N*. *crassa*, and unlike *Saccharomyces*, the two paralogs have some distinct functions.

### CSP-6 associates with the WCC and acts as a phosphatase on WC-1

**Fig 2:**
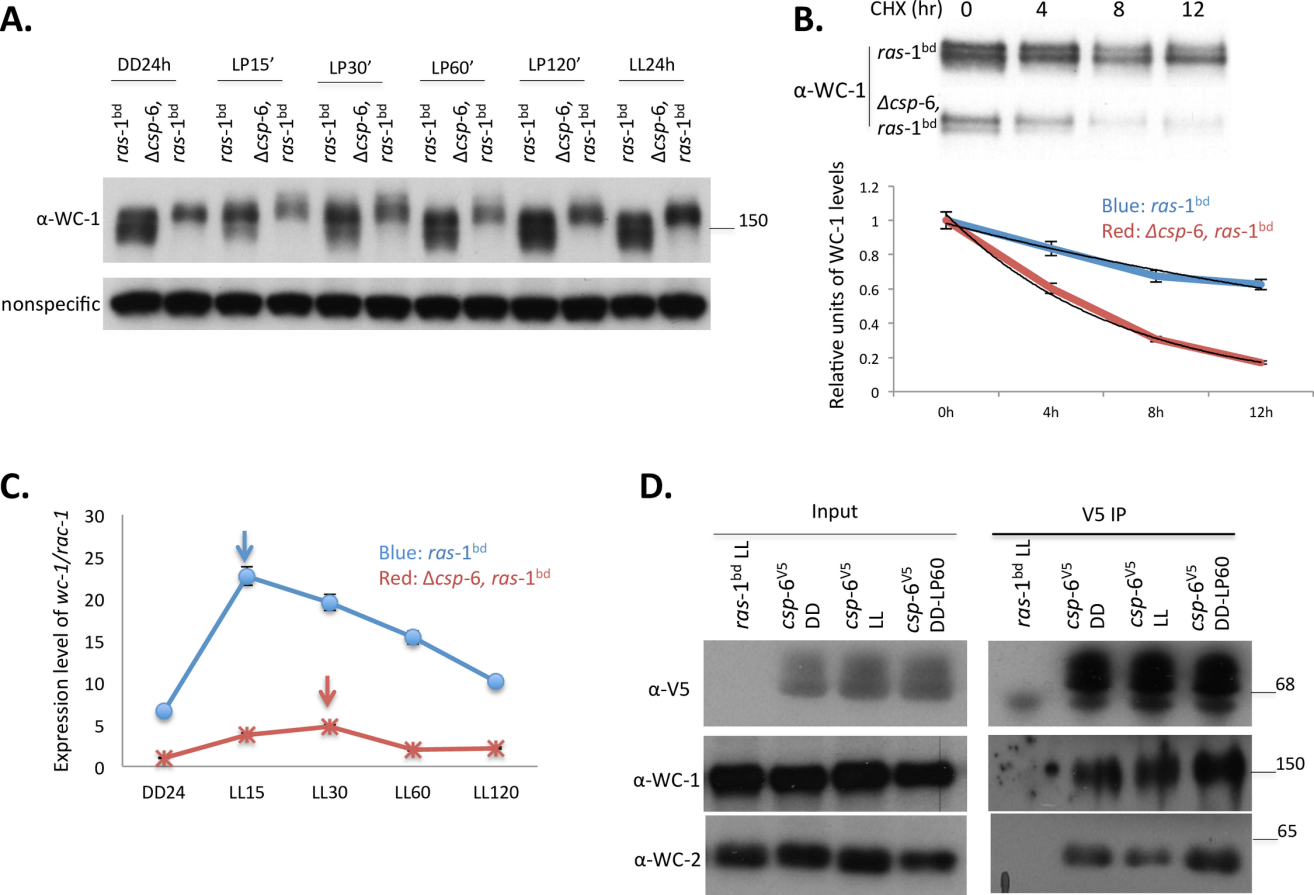
CSP-6 interacts with WC-1 to regulate its phosphorylation status, stability and expression. A: Western blot analysis showing the amount and degree of modification of WC-1 in *ras*-1^bd^ and Δ*csp-*6, *ras*-1^bd^ strains. Nonspecific bands were shown for equal loading. The cultures were harvested at the indicated time points. (DD24: constant darkness for 24h; LP15-120’: Light pulse for 15-120min after DD24; LL: constant light. B: WC-1 is less stable absent CSP-6. Representative Western blots of WC-1 following addition of CHX show the rate of WC-1 degradation (Upper). Results of densitometric analysis (Lower) for *ras*-1^bd^ (blue) and Δ*csp*-6, *ras*-1^bd^ (red) +/- SD. C: Light-induction of *wc-*1 is reduced in Δ*csp*-6. RNA was isolated from *ras*-1^bd^ and Δ*csp*-6, *ras*-1^bd^, and amounts of *wc*-1 mRNA quantified by qRT-PCR. D: Co-IP demonstrating physical interaction between CSP-6 and WC-1 *in vivo*. CSP-6 was tagged with a V5 epitope at its C-terminal and immunoprecipitation was performed using V5 agarose beads. Western blot of V5-purified CSP-6 shows WC-1 was pulled down in all indicated conditions together with WC-2. Protein maker(s) were shown as with the molecular weight (KDa) as indicated.

Because the WCC drives *frq* expression and we observed delayed transcriptional and translational rhythms of *frq*/FRQ (Fig 1C–1E), we examined the expression of WC-1. As shown in Fig 2A, the protein levels of WC-1 in DD (constant dark), LL (constant light) or following a light pulse (LP) were significantly reduced in Δ*csp*-6, and WC-1 was hyperphosphorylated in Δ*csp*-6 in all conditions we examined (Fig 2A). To confirm that the mobility shift of WC-1 in Δ*csp*-6 is caused by phosphorylation, total protein of wild type and Δ*csp*-6 were treated with λ-phosphatase. The results showed that WC-1 in both wild type and Δ*csp*-6 collapsed to the same level in both tested time points, constant dark 24h and light pulse 15min, indicating the low mobility form of WC-1 in Δ*csp*-6 is a hyperphosphorylation form of WC-1 (S3A Fig).

In addition, WC-1 is less stable in Δ*csp*-6 than in wild type indicating that CSP-6 stabilized the WC-1 protein (Fig 2B). Furthermore and consistent with Western analysis, *wc-*1 mRNA levels were significantly reduced in Δ*csp*-6 under all conditions as well, and, although *wc*-1 is still light-induced, it took longer (LP30 min) to reach peak in the Δ*csp-*6, indicating that the light response was affected in *Δcsp-*6 (Fig 2C). WC-1 is required for light-induced carotenogenesis and cultures of Δ*csp-*6 were pale pink instead of orange, so we tested expression level of three *albino* genes in the Δ*csp-*6 mutant (S3B Fig). All three *albino* genes (*al*-1, *al-*2, *al-*3) showed reduced mRNA levels in Δ*csp*-6 consistent with reduced carotenoid accumulation and the pale color in Δ*csp-*6 (S3C Fig). Additionally, we examined the mRNA expression level of a few light induced genes *al*-1, *al*-3, *sub*-1 and *frq*, following light pulses in order to understand whether hyperphosphorylated WC-1 in Δ*csp-*6 would affect their light response. Interestingly, *sub*-1 and *frq* showed normal kinetics in Δ*csp-*6 while *al-*1 and *al*-3 showed a delayed light response compared to the wild type, and *al*-1, *al*-3, *sub*-1 all had reduced expression in Δ*csp-*6 (S3D Fig). These data suggested that the inability to dephosphorylate WC-1 in Δ*csp-*6 impacted expression its downstream targets.

The hyperphosphorylation of WC-1 observed in the Δ*csp*-6 background suggested that WC-1 could be a direct target of CSP-6. To determine whether CSP-6 interacts with WC-1, co-immunoprecipitation (co-IP) assays were performed using CSP-6 epitope tagged with V5 on its C-terminus, under conditions of DD, LL and LP60min. The data show that CSP-6 can interact with WC-1 to regulate WC-1 phosphorylation under all tested conditions (Fig 2D).

Additionally, although we noticed a reduced level of WC-2 in Δ*csp-*6, the phosphorylation level of WC-2 was not significantly changed in the Δ*csp-*6 mutant indicating that CSP-6 more specifically targets WC-1 (S3E Fig). Interaction was also detected between CSP-6 and WC-2 (Fig 2D), although we expect this is most likely indirect, reflecting the interaction between CSP-6 and WC-1 which heterodimerizes with WC-2.

### Structure function analysis of CSP-6 shows the HAD domain is sufficient partially to rescue output defects in a manner dependent on glucose concentration

**Fig 3:**
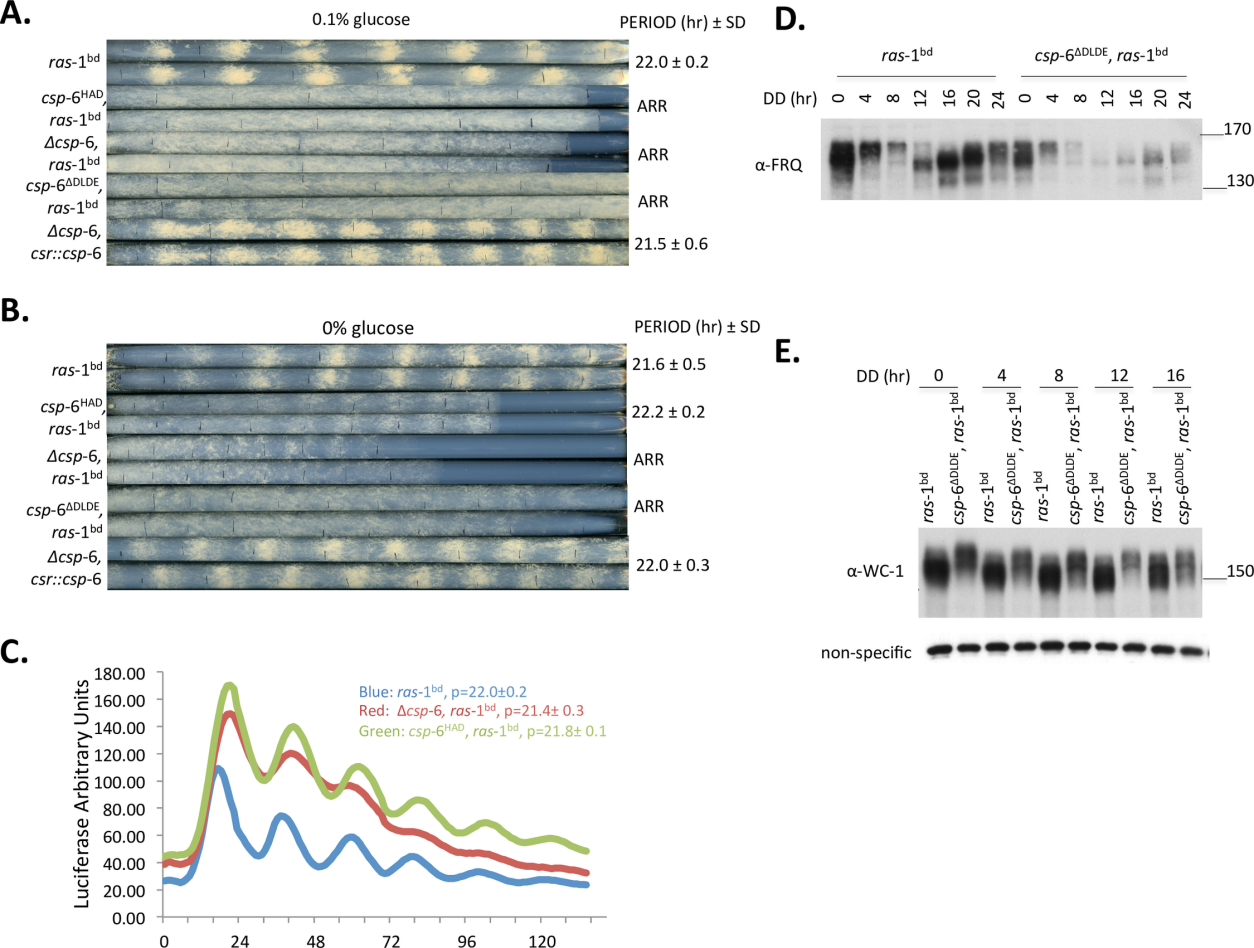
The catalytic HAD phosphatase domain can partially rescue Δ*csp-6* phenotypes dependent on glucose concentration. A: Race tube assays of wild type *ras-*1^bd^; Δ*csp-*6, *ras*-1^bd^; Δ*csp-*6, *csr*::*csp-*6^HAD^,*ras*-1^bd^; Δ*csp-*6,csr::*csp-*6^ΔDLDE^, *ras*-1^bd^; and Δ*csp*-6, *csr*::*csp*-6, *ras*-1^bd^(a full-length *csp-*6 complementary strain) on race tube medium containing 0.1% glucose. All complementation constructs were transformed into Δ*csp-*6 by targeting to the *csr* locus. Duplicate race tubes for each strain are shown. Period is reported in hours ± one standard deviation; ARR is arrhythmic. B: Race tube assay for the same sets strains on race tube medium without glucose, showing *csp-*6^HAD^ expresses conidiation rhythms after 2-day under free running in constant darkness. C: Analyses of *frq* transcriptional rhythmicity in, Δ*csp*-6, *ras*-1^bd^ and *csp*-6^HAD^, *ras*-1^bd^ by luciferase activity. ‘p’ stands for period. D: Western blot analysis showed that FRQ protein remains rhythmic in *csp-*6^ΔDLDE^, *ras*-1^bd^. Cultures were harvested in constant darkness at the indicated time. E: Western blot analysis showing hyperphosphorylated WC-1 in *csp-*6^ΔDLDE^, *ras*-1^bd^ strains at the indicated times in darkness. Protein maker(s) were used with the molecular weight (KDa) as indicated.

In CSP-6, only the HAD phosphatase domain is predicted to dephosphorylate its substrates (S1A Fig). To determine whether the HAD domain was sufficient to rescue defects caused by deletion of *csp-*6, we transferred a construct bearing only the HAD domain (*csp-*6^HAD^, amino acids 222–388, under the control of 1.5 kb of the native *csp-*6 promoter) to the *csr* locus. The resulting transformants failed to produce overt rhythmicity on race tubes while the full length *csp-*6 construct successfully complemented the banding defect though with slightly reduced growth rate (Fig 3A). To exclude the possibility that the N-terminal region of *csp-*6 might be sufficient for circadian function, we also generated construct *csp-*6^ΔHAD^ (amino acids 1–221), lacking the HAD domain, and transformed it into Δ*csp*-6. The resulting transformants were as arrhythmic as Δ*csp*-6 on race tubes.

However, one interesting phenomenon emerged when transformants were examined under conditions of glucose depletion. After a few days, transformants bearing *csp-*6^HAD^ showed weak conidiation rhythms whereas no banding was detected in Δ*csp*-6 under the same conditions (Fig 3B). To confirm core clock function in *csp*-6^HAD^ we used the *frq c-box-luc* transcriptional reporter, showing that *csp*-6^HAD^ had the delayed phase seen with Δ*csp*-6 but with more robust rhythmicity than that of Δ*csp*-6. (Fig 3C). These results indicated that the HAD domain itself could partially rescue clock defects caused by loss function of CSP-6 under tested conditions.

HAD phosphatases, which have essential Asp residues in their catalytic domains, are emerging as a large family existing in plants, prokaryotes and mammals. Their conserved active sites have a consensus sequence hhhDxDx (T/V)(L/V) h, where h represents a hydrophobic residue, and x indicates any amino acid [38], the two aspartates coordinating the essential Mg^2+^ in the active site. In the yeast, mutation of DXDX(T/V) motif cannot functionally complement the *psr1/psr2* mutant. It is essential for its sodium stress response suggesting that mutation of DXDX disrupted its phosphatase activity [37]. To investigate if the DxDx motif (DLDE) in CSP-6 that is essential for phosphatase activity is also essential for its circadian function (S2A Fig), we generated a *csp*-6^ΔDLDE^ construct and transferred it into the *csr* locus of a Δ*csp*-6 mutant. Race tubes of *csp-*6^ΔDLDE^ failed to show overt rhythmicity with or without glucose (Fig 3A and 3B). Western blot analyses showed that compared to the wild type, FRQ protein along with phosphorylation level of *csp*-6^ΔDLDE^ was rhythmic as that shown in Δ*csp*-6 (Fig 3D). However, similar to Δ*csp*-6, the FRQ level *csp*-6^ΔDLDE^ was reduced significantly after moving to dark, which is consistent to the lower level WC-1 in *csp*-6^ΔDLDE^ (Fig 1D, Fig 3D and 3E). Additionally, we detected hyperphosphorylated WC-1 in *csp*-6^ΔDLDE^ under constant dark and light (Fig 3E, S3A Fig). These data confirmed that the conserved DLDE motif essential for phosphatase activity was critical for CSP-6 circadian activity.

### CSP-6 interacts genetically with VVD and reduces the physical interaction between VVD and WC-1

**Fig 4:**
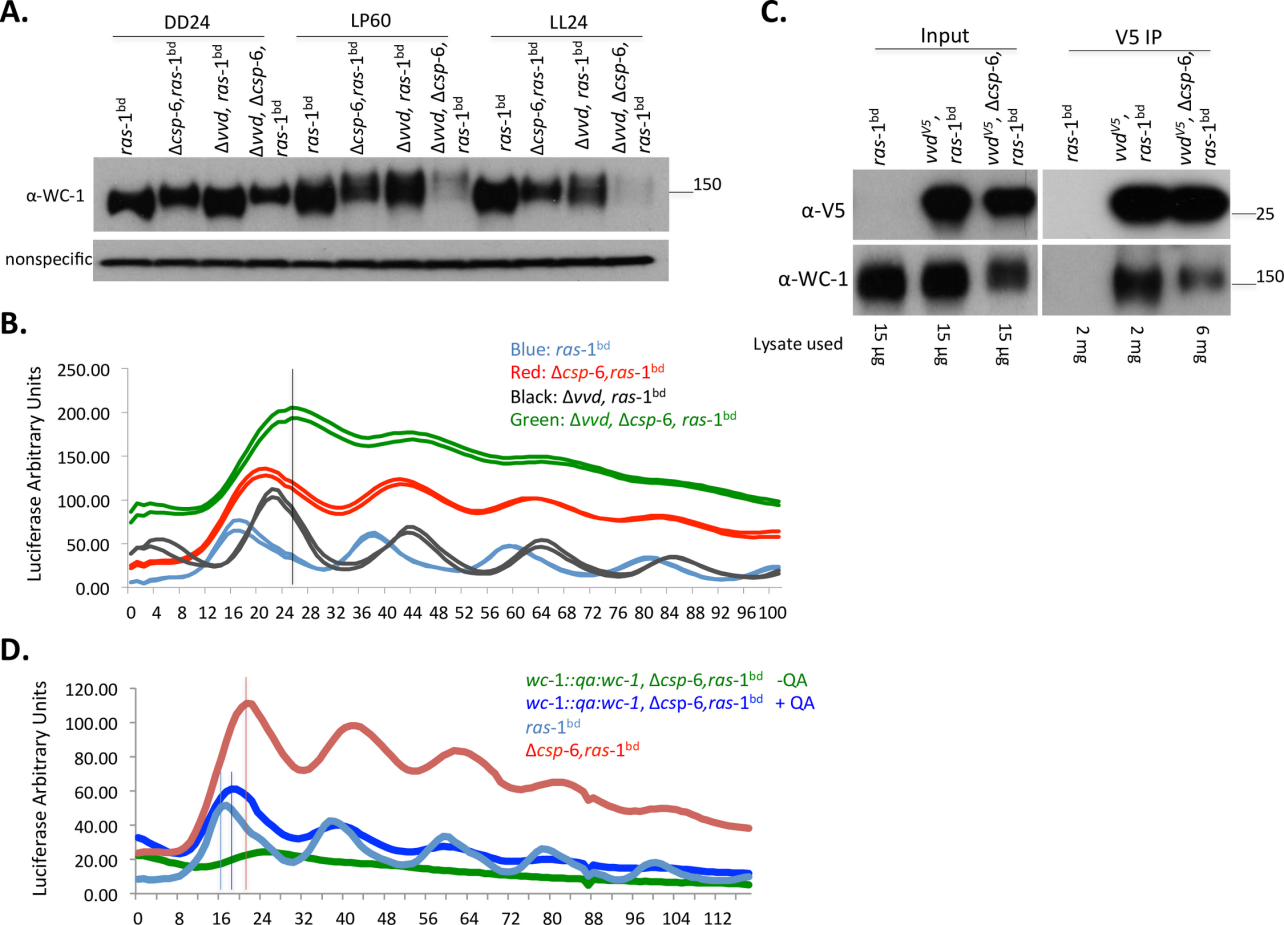
Reduced interaction between WC-1 and VVD in the Δ*csp-6* mutant affects WC-1 phosphorylation and the phase of *frq* expression. A: Western blot analyses showing WC-1 levels in *ras*-1^bd^; Δ*csp-*6 *ras*-1^bd^, Δ*vvd*, *ras*-1^bd^ and Δ*csp-*6, Δ*vvd*, *ras*-1^bd^ under indicated conditions. B: Representative luciferase traces of *frq c-box-luc* showing a further phase delay in the Δ*csp-*6, Δ*vvd* double mutant as compared to either single mutant. C: VVD-WC-1 interaction at LP60min in both *vvd*^V5^ and *vvd*^V5^,Δ*csp*-6 in the *ras*-1^bd^ background; *ras*-1^bd^ was used as negative control. VVD-V5 was immunoprecipitated using anti-V5 agarose beads. Immunoprecipitates were analyzed by Western blot using WC-1 antibody. Because reduced WC-1 was detected in the Δ*csp-*6 mutant, we used three times more extract for the IP of *vvd*^V5^, Δ*csp-*6 (6mg) than those of wild type (2mg) background, based on the quantification by Western blot (S4A Fig) to make sure similar amounts of WC-1 were available for IP. D: Representative luciferase activity assays of *frq-luc* in strain *wc*-1::*qa*:*wc-*1, Δ*csp*-6 in the presence or absence of 0.01M QA, showing that increased WC-1 levels can partially rescue the phase delay phenotype of Δ*csp*-6.

The Δ*csp*-6 strain displayed a four-hour phase delay, as well as a reduced level of WC-1 that is hyperphosphorylated (Figs 1 and 2), all similar to phenotypes observed in the Δ*vvd* mutant [26]. Evidence for genetic interaction between *csp-*6 and *vvd* was seen when Δ*csp*-6, Δ*vvd*, *ras-*1^bd^, *csr*::*c-box-luc* cultures growing on minimal slants showed enhanced carotenoid accumulation (S3B Fig), and this was confirmed at the protein level when Western blot analyses showed much less WC-1 in the double mutant especially in the light, indicating that deletion of *csp-*6 and *vvd* together had synergistic effects compared to the individual mutants (Fig 4A). Surprisingly, even with dramatically reduced WC-1 levels, the luciferase activity driven by *frq* c-box in the Δ*csp*-6, Δ*vvd* double mutant appeared robust. Furthermore, a nearly 8 hr phase delay was detected in the double mutant and rhythmicity was dampened after three cycles, a plainly additive or synergistic effect as neither was observed in the single mutants Δ*csp*-6 or Δ*vvd* (Fig 4B). These data suggest that CSP-6 and VVD contribute to the separate but parallel pathways, based on the synergistic phenotype of double mutants.

VVD governs photoadaptation and influences light responses [26] and does so by interacting physically with WC-1 [11]. Because weaker light-induction and delayed photoadaptation was detected in the Δ*csp*-6 mutant (Fig 2C), we hypothesized that a disruption of the interaction between VVD and WC-1 could be a contributing factor. To test this, we used a *vvd*^V5^, Δ*csp*-6 strain and performed a Co-IP assay between VVD and WC-1, adjusting the input amounts to 3 times (6mg) more than that used in the wild type (2mg) so that equivalent WC-1 was present in all samples (Fig 4C, S4A Fig). These data indicate a substantial reduction in the amount of WC-1 interacting with VVD in the Δ*csp*-6 mutant compared to wild type, even with comparable amounts of WC-1 input (Fig 4C). This reduced interaction between VVD and WC-1 might result in the weaker light response seen in the Δ*csp*-6 mutant, and combined with the reduced level of WC-1 seen in Δ*csp*-6 could also underlie the additive phase delay.

To test this hypothesis we used a strain in which *wc-*1 expression was driven by the inducible *qa-2* promoter at the native *wc-1* locus [14] in the Δ*csp*-6, *ras-*1^bd^, *csr*::*C-box*-luc background. Under quinic acid (QA) induction (10^-2^M QA), high levels of WC-1 comparable to wild type can be expressed constitutively in the Δ*csp*-6 mutant (S4B Fig) and hyperphosphorylated WC-1 can be detected as well in the *qawc*-1, Δ*csp*-6 strains. The *wc-*1::*qa*:*wc*-1, Δ*csp*-6 strain was arrhythmic absent inducer and rhythmic with inducer for the first 3 cycles and the enhanced WC-1 expression in *wc-*1::*qa*:*wc-*1, Δ*csp*-6 substantially rescued the phase delay phenotype, reducing the 4 hr phase delay to around two hours (Fig 4D). To exclude the possibility that quinic acid caused the dampening in *wc-*1::*qa*:*wc-*1, Δ*csp*-6, we also tested the rhythmicity of *wc-*1::*qa*:*wc-*1 grown with 10^-2^M QA; the results showed that *wc-*1::*qa*:*wc-*1 displayed robust rhythmicity through tested 5 days (S4C Fig). Although the QA-induced increase in WC-1 protein level in the Δ*csp*-6 mutant largely rescued the phase delay phenotype, race tube analysis showed that QA-induced WC-1 failed to rescue the overt conidiation rhythm in Δ*csp*-6 (S4D Fig), indicating that downstream WCC targets or certain clock-control genes regulating circadian output were misregulated in the Δ*csp*-6 mutant and this most likely was caused by the hyperphosphorylated WC-1 but not the reduced WC-1 amount.

### CSP-6 forms a complex with WHI-2 and plays a major role in the complex

**Fig 5:**
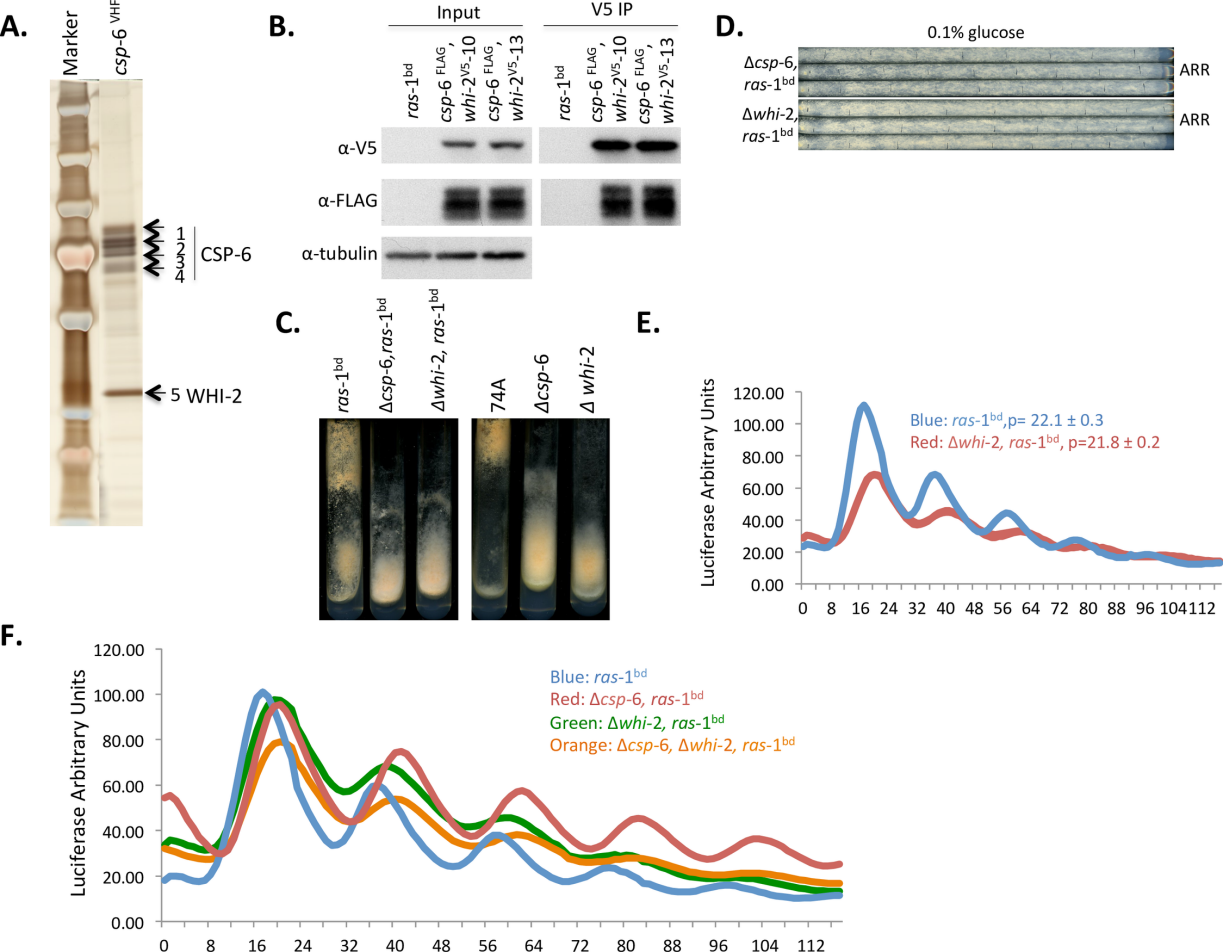
CSP-6 associates with WHI-2 *in vivo*, and loss of *whi*-2 results in phenotypes similar to Δ*csp*-6. A: Silver stained SDS-PAGE gel showing CSP-6 associated with WHI-2 (band 5) as confirmed by MS-based protein sequencing. Bands 1–4 correspond to CSP-6 itself based on Mass Spec data (S2 Table). B: Co-immunoprecipitation assay showing CSP-6 physically interacts with WHI-2 *in vivo*. A wild type strain (*ras*-1^bd^) that lacks the epitope tag was used as a negative control. Two individual transformants (-10 and -13) of *csp-*6^FLAG^, *whi*-2^V5^ were used in Co-IPs and Western analyses. C: Cultures of WT (*ras*-1^bd^ or 74A), Δ*csp-*6 and Δ*whi*-2 (with or without *ras*-1^bd^ respectively) were grown on solid minimal slants showing similar growth morphology between Δ*csp*-6 and Δ*whi-*2. D: Race tube data showing loss of overt conidiation rhythms in Δ*whi*-2; triplicates of each strain are shown. E: Luciferase traces showing *frq-luc* was circadianly rhythmic but expressed with a delayed phase in the Δ*whi*-2 mutant. ‘p’ stands for period. F: Luciferase activity assay of *frq C-box-luc* in strains of *ras-*1^bd^, the Δ*csp*-6, *ras-*1^bd^ strain, the Δ*whi-*2, *ras*-1^bd^ strain, and double mutant Δ*csp*-6, Δ*whi*-2, *ras-*1^bd^ strain, showing the phase delay seen in Δ*csp-*6 and in the double mutant Δ*csp-*6, Δ*whi-*2 was similar.

To identify CSP-6-associated proteins, CSP-6 was epitope tagged with VHF (V5, His and FLAG tandem tag) and used to purify CSP-6 by tandem affinity purification using FLAG agarose followed by V5 magnetic beads. Mass spectrometric analysis showed that the four bands (labeled as 1–4 from silver staining) clustered together around 70kDa are all CSP-6, and the one band below was identified as a weak ortholog (BLASTP e-11) of *Saccharomyces* WHI-2 encoded by NCU10518 (Fig 5A). Two translational start sites were found in the *csp*-6 5’UTR suggesting that CSP-6 has two protein isoforms with a size difference of 5.5 kDa (S5A Fig). We also performed phosphatase treatment on V5-purified CSP-6 and the results showed that the proteins can apparently be dephosphorylated indicating that, like its Saccharomyces ortholog Psr1p, post-translational modification occurs to CSP-6 after it is synthesized (S5B Fig).

*Saccharomyces* Whi2p is a general stress regulator protein known to interact physically and genetically with Psr1p and Psr2p, the ortholog and paralog of CSP-6 respectively, and is believed to activate them; knockouts of any members of the Psr(s)/Whi-2 complex in yeast share similar phenotypes [39,40]. We confirmed the interaction between CSP-6 and WHI-2 in *Neurospora* by performing Co-IP (Fig 5B), and then used a Δ*whi*-2 strain obtained from the *Neurospora* knockout collection [41] to ask whether WHI-2 played a role in the circadian system. Δ*whi-*2 showed growth, morphology, and circadian phenotypes similar to Δ*csp-*6 including slow growth, reduced conidiation, circadian output defects on race tubes, a phase delay in the core clock (assayed by *frq c-box-luc* reporter), and increased WC-1 phosphorylation level (Fig 5C–5E, S6A Fig). However, in most cases the defects in Δ*whi*-2 were not as severe and were in all cases hypostatic to those seen in Δ*csp-*6: e.g., the phase delay in Δ*whi-2* was not as severe as Δ*csp-*6 (Fig 5F), and WC-1 protein amounts were not reduced significantly in the Δ*whi-2* mutant (S6A Fig). The double mutant Δ*csp*-6, Δ*whi-*2 had WC-1 levels similar to Δ*csp-*6, and no further hyperphosphorylation of WC-1 was detected in the Δ*csp*-6, Δ*whi-*2 double mutant (S6B Fig) compared to Δ*csp*-6. These results suggest that in the CSP-6/WHI-2 complex, CSP-6 plays a major role in regulating circadian related phenotypes including phase and conidiation rhythmicity, while WHI-2 is more like an assistant to fully activate CSP-6.

### WCC-mediated circadian and light regulation of *adv-1* is abolished in the Δ*csp-6* mutant

**Fig 6:**
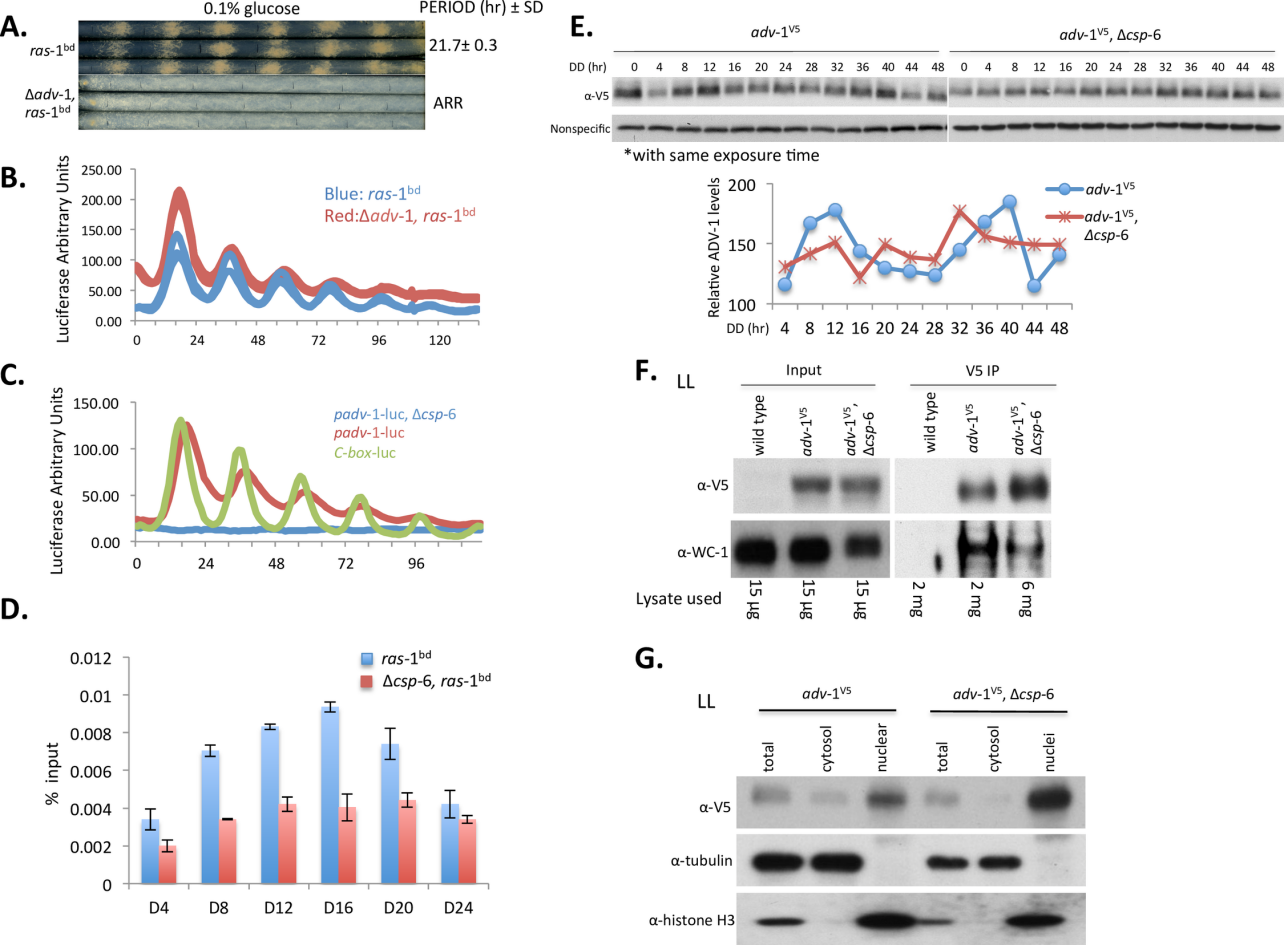
ADV-1 is a direct downstream target of CSP-6-WC-1 that regulates circadian output. A: Race tube data showing loss of overt conidiation rhythmicity in Δ*adv*-1; triplicate race tubes are shown. Period is reported in hours ± one standard deviation, ARR is arrhythmic. B: Luciferase traces of *frq c-box-luc* in the *ras-*1^bd^ and Δ*adv-*1, *ras-*1^bd^ genetic backgrounds confirming that the clock functions normally in Δ*adv*-1. C: Representative luciferase activity assays showing circadian rhythmicity of *adv*-1 promoter activity was significantly impaired in the Δ*csp*-6 mutant. D: ChIP assays were performed in a time course across a circadian cycle from DD4 to DD24 to show the recruitment of WC-2 to the *adv*-1 promoter in the *ras-*1^bd^ and Δ*csp-*6, *ras-*1^bd^. Error bars reflect +/- 1SD, n = 3. E: Western blot analysis of protein expression of V5-tagged ADV-1 in WT and Δ*csp-*6 (Upper). The data shows protein ADV-1 is expressed rhythmically in the wild type strains but the rhythmicity was lost in Δ*csp-*6. Densitometric analysis of these data is shown in the lower panel. F: Co-IP assay demonstrating ADV-1 and WC-1 interaction using DSP crosslinking in constant LL. V5-tagged ADV-1 was co-immunoprecipitated with anti-V5 agarose beads and WC-1 was detected with anti-WC-1 antibody. To compensate for the reduced WC-1 present in Δ*csp*-6, 3 fold more lysate was used for the V5-IP in *adv*-1^V5^, Δ*csp*-6 as compared to *adv*-1^V5^; even so, reduced interaction between ADV-1 and WC-1 was detected in Δ*csp-*6 compared to WT. G: ADV-1 protein is enriched in nuclei. Subcellular fractions from *adv*-1^V5^ in WT and Δ*csp-*6 were analyzed with the indicated antibodies. Tubulin was used as a control to show nuclear fraction was not contaminated with cytosolic protein and histone H3 was used to show enrichment of the nuclear fraction.

Although driving WC-1 protein amounts to wild type levels can partially rescue the phase delay phenotype of Δ*csp-*6 mutant (Fig 4D), it failed to rescue circadian rhythmicity on the race tubes (S4D Fig). These data indicated a break in circadian output control downstream from the WCC in the absence of CSP-6, so we sought the source of the break in control. Because in *Saccharomyces* Psr1p functions together with Whi2p to activate stress responses and mediate gene expression through the stress-responsive transcription factor MSN2p [39], we asked whether the ortholog of yeast MSN2 in *N*. *crassa*, that is MSN-1, a cutinase G-box binding protein encoded by NCU02671, was involved in clock output. The Δ*msn-1* mutant, however, displayed normal circadian banding and period length (though with a reduced growth rate; S7A Fig) and *frq c-box-luc* reporter assays showed that Δ*msn-*1 had a robust circadian rhythm (S7B Fig), all data indicating that in *Neurospora* CSP-6 does not act through *msn-*1 to regulate circadian output. Because Ghosh et al (2014) had suggested that *csp*-6 might act in the same pathway as *csp*-1 [42] to regulate growth and conidiation, we also confirmed that transcriptional rhythmicity of *csp*-1 was not affected in the Δ*csp-*6 mutant (S7C Fig). After this and following the genetic principle of epistasis, we screened transcription factors known to be targets of the WCC [21,22] for circadian output defects, showing regulation of *fluffy* (a major regulator of conidiation) to be still weakly rhythmic in Δ*csp-*6 (S7D Fig) before rediscovering that deletion of *adv-1* (NCU07392) results in defects similar to Δ*csp-6* (Fig 6A) [22]. Though deletion of *csp*-6 did not affect the oscillation of *csp*-1 and *fluffy* promoter activity, we found that the transcriptional expression level of *csp*-1 and *fluffy* was reduced in Δ*csp-*6 compared to that in the wide type (S7E Fig).

ADV-1 is a transcription factor previously shown broadly to affect development [41], to be robustly regulated by light [21] and the clock, and to be required for the overt rhythm in conidiation on race tubes [22,33]. Consistent with prior data [22] the *frq-luc* reporter remained robustly rhythmic in Δ*adv-*1 indicating that ADV-1-regulated circadian output does not impact the core oscillation (Fig 6B) but importantly, by comparing *adv-*1 transcriptional activity in wild type and Δ*csp-*6 backgrounds, it is clear that the normally rhythmic transcription of *adv*-1 is lost in Δ*csp*-6 (Fig 6C).

Because *adv-*1 is a direct downstream target of WC-1 [22], we hypothesized that the arrhythmicity of *adv*-1/ADV-1 in Δ*csp-*6 might be caused by the misregulation of WCC binding efficiency at the *adv-*1 promoter. ChIP assays using WC-2 antibody were performed across a time course from 4h to 24h in darkness. The results showed that rhythmic WCC-binding at *adv*-1 promoter sites was disrupted in the Δ*csp-*6 mutant as compared to wild type (Fig 6D). Binding was also attenuated following light pulses (LP15min) (S8A Fig). The loss of circadian regulation of ADV-1 in Δ*csp-*6 was also confirmed at the protein level (Fig 6E) demonstrating that CSP-6 is essential for rhythmicity of *adv-*1/ADV1-1 at both transcriptional and translational levels.

Because of the disruption of WCC binding to the *adv*-1 promoter elicited by loss of *csp*-6 we asked whether there was a corresponding disruption of oscillator-relevant binding to the C-box within the *frq* promoter in Δ*csp-*6 (S8B and S8C Fig); overall binding was reduced roughly half in first 24h in darkness and even more significantly reduced WCC binding was detected in day two from 28-48h in darkness. Consistent with the *frq*-luc luciferase data in Fig 1B, deletion of *csp*-6 did not affect the rhythmicity of FRQ, but its robust expression. These data again suggest that the phosphorylation status of WC-1 as impacted by loss of CSP-6 has a discrete effect on circadian output, and moreover the effect may not be only on the ability of WC-1 to bind to the *adv*-1 promoter but also on its ability to activate expression from it.

We asked separately whether CSP-6 can physically interact with ADV-1 to regulate its transcriptional and translational activity and confirmed via pull down assays using an epitope tagged *adv*-1^V5^, *csp-*6^FLAG^ strain that no direct interaction between CSP-6 and ADV-1 was detected under the conditions used (S9A Fig). In addition, CSP-6 does not regulate the light response of ADV-1 though slightly reduced ADV-1 protein level was detected in Δ*csp-*6 (S9B Fig). A dephosphorylation assay was also performed to further confirm that CSP-6 did not function on ADV-1 directly as a phosphatase (S9C Fig). Taken altogether these data are most easily interpreted as showing that hyperphosphorylation of WC-1 caused by loss of CSP-6 reduced the binding efficacy of the WCC at the *adv-*1 promoter and that CSP-6 regulates circadian output and light-regulation via impacting transcription of *adv*-1 through the WCC. However, in addition to disruption of rhythmic WCC binding to the *adv*-1 promoter, reduced interaction between ADV-1 and WC-1 was also detected by Co-IP (Fig 6F); these experiments used three times more IP protein from the *adv-1*^V5^, Δ*csp-6* (6mg) strain as compared to *adv-1*^V5^ (2mg) to make up for the reduced WC-1 expression seen in *adv-*1^V5^, Δ*csp*-6. This result confirmed that loss of *csp-*6 substantially interrupted the interaction between WC-1 and ADV-1.

We then examined the subcellular distribution of ADV-1 in cultures grown in LL. Aliquots of total, cytosol and nuclear fractions were analyzed by Western blotting (Fig 6G). Two proteins, tubulin and histone-H3 were used as cytoplasmic and nuclear protein markers, respectively, to confirm the quality of the nuclei and to control for cytoplasmic contamination in the nuclear preparation. ADV-1 was enriched in nuclei, which was consistent with its function as a transcription factor (Fig 6G). Interestingly however, although slightly less total ADV-1 was detected in Δ*csp-*6, more of it was enriched in nuclei, suggesting that loss of *csp-6* resulted in mildly misregulated localization such that ADV-1 was more strongly partitioned to nuclei rather than to the cytoplasm. Furthermore, the Δ*csp-*6 mutant displayed only slightly less ADV-1 protein and no difference in ADV-1 phosphorylation as compared to WT, suggesting that CSP-6 did not directly work on ADV-1 as a phosphatase (S9B Fig). Therefore, the reduced interaction between ADV-1 and WC-1, and increased amount of nuclear ADV-1 in Δ*csp-*6 (Fig 6F and 6G) may reflect the action of CSP-6 on WC-1 rather than directly on ADV-1.

### CSP-6 is found in the nucleus

**Fig 7:**
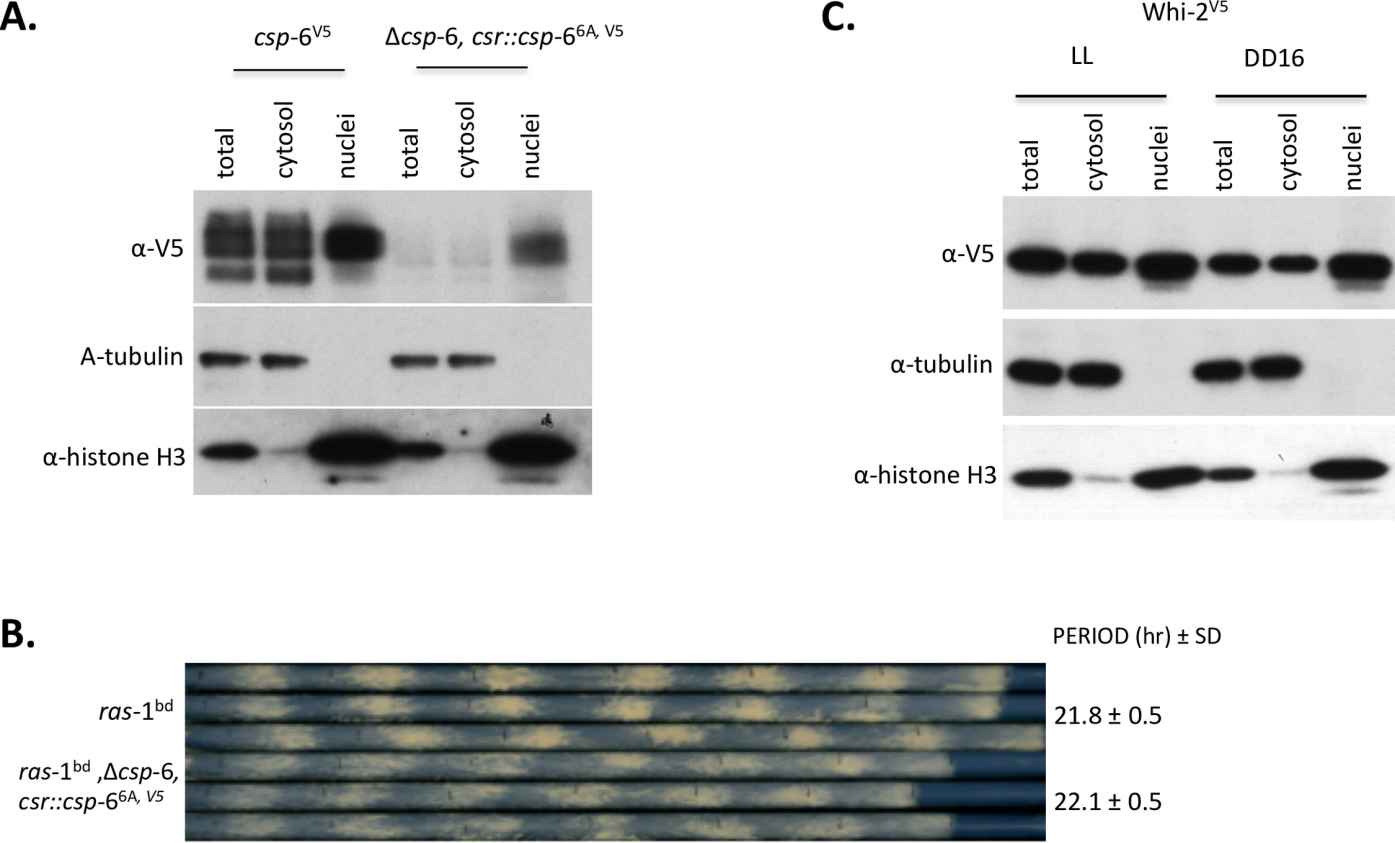
CSP-6 localizes inside nuclei in *Neurospora*. A: Western blots show CSP-6 and two control proteins (tubulin and histone-H3) in total cell lysates (Total), cytoplasm (Cyto) and nuclear fractions of *csp*-6^V5^ and Δ*csp*-6, *csr*::*csp-*6^6A, V5^. CSP-6 was detected in the nuclei; there was no tubulin signal in the nuclear fraction suggesting that isolated nuclei have little cytosolic contamination. B: Race tube assay showing Δ*csp*-6 *ras*-1^bd^; *csr*::*csp*-6^6A, V5^ has normal overt circadian rhythmicity with the same period as wild type (*ras*-1^bd^); triplicate race tubes are shown. Period is reported in hours ± one standard deviation, ARR is arrhythmic. C: Subcellular distribution of WHI-2 was analyzed by Western blot with indicated antibodies. Cellular fractions were prepared by differential centrifugation by standard techniques (57), but the final nuclear pellet was resuspended in just 300 μl of buffer versus 40 ml for total and cytoplasmic fractions. In all cases the same volume of extract (10μl) was loaded on the gels, so estimates of the total amount of protein in each compartment must reflect both the amount seen on the gel and the total amount of extract prepared.

As reflected in the gene name, *Saccharomyces* PSR1 (Plasma membrane Sodium Response 1) localizes to the plasma membrane [37,39] and we were interested to know whether CSP-6 has similar localization in *Neurospora*. Nuclei were isolated from *csp*-6^V5^, *ras*-1^bd^ and the cytoplasmic and nuclear fractions analyzed by SDS-PAGE [43] (Fig 7A). CSP-6 was enriched in the nuclear fraction but was still present in the cytoplasm. Therefore, unlike yeast, *Neurospora* CSP-6 localized to both the cytoplasm and nucleus.

Sequence analysis of CSP-6 revealed a putative nuclear localization signal ‘PKKKKG’ (9-14aa) near the N-terminus that is absent from yeast Psr1p (S10 Fig). To determine whether ‘PKKKKG’ is a nuclear localization signal (NLS) in CSP-6, ‘PKKKKG’ was replaced by ‘AAAAAA’ (6A) and the construct (including a C-terminal V5 tag) transformed into the *csr* locus of Δ*csp*-6 driven by its own promoter. The resulting transformant (Δ*csp-*6, *csr*:: *csp-*6^6A, V5^) rescued the banding defect and growth rate of the Δ*csp-*6 mutant (Fig 7B); however, ‘PKKKKG’ with 6A does not abolish the nuclear localization of CSP-6 indicating that PKKKKG is not a NLS (Fig 7A). In addition, we noticed that CSP-6 was reduced significantly in amount in Δ*csp*-6, *csr*:: *csp-*6^6A, V5^ suggesting PKKKKG may be essential for CSP-6 stability though not as a NLS. Cellular fractionation showed that WHI-2 displayed localization similar to that of CSP-6 (Fig 7C).

## Discussion

*csp-6* encodes a phosphatase required for *Neurospora*’s major overt circadian output, the daily cycle of asexual development. However, CSP-6 is constitutively expressed over the day without obvious rhythmicity and is thus not a typical clock-controlled gene [30]. Previous studies associated two protein phosphatases PP1 and PP2A, with the *Neurospora* core circadian oscillator, PP1 implicated in regulating stability of FRQ and PP2A impacting negative feedback and acting on WC-1 *in vitro* [18]. CSP-6 plainly has a major effect on WC-1 phosphorylation; however, deletion of *csp*-6 has relatively less effect on core clock oscillations (a reduced amplitude and delayed phase but normal period length), instead only having a significant discrete effect on circadian output including phase setting and conidiation rhythms. Because of the high likelihood that WC-1 appears to be a direct target of CSP-6 *in vivo*, interpretation of its actions is revealing both in the context of the oscillator and of output and these are addressed sequentially.

The *Neurospora* blue light photoreceptor and clock protein WC-1, in association with WC-2, regulates expression and oscillation of FRQ. FRQ then undergoes a cycle of phosphorylation that eventually impacts its ability to interact with WCC [44]. Biochemical analyses of cell extracts have suggested that dephosphorylation of WCC may enhance its DNA binding activity to the *frq* promoter [13,2,24]; however, these effects could be mediated by other proteins in the extracts such as FRQ whose phosphorylation status affects WCC’s activity. Similarly, hyperphosphorylated WCC observed in cell extracts of phosphatase mutants including PP1, PP2A was reported to have a reduced binding activity to the *frq* promoter, although again is not possible to say whether the effect on WCC is direct or via interacting proteins. In another correlation, activation of WCC was reported to be dependent on RGB-1, a regulatory subunit of PP2A, and was correlated with dephosphorylation of WC-1 and WC-2, data supporting a model in which the rhythmic activity of WCC is controlled by a dynamic equilibrium of phosphorylation and dephosphorylation mediated by several kinases and phosphatases [45]. In all these studies, then, a correlation was developed between phosphorylation of WCC and reduced activity of WCC. However, direct *in vivo* interaction between these phosphatases and WC-1 has not been demonstrated, correlation does not establish cause and effect, and data presented here are not entirely consistent with this model for WC-1 regulation. We saw significantly hyperphosphorylated WC-1 in the strain lacking CSP-6, a phosphatase protein that interacts with WC-1 (Fig 2). This suggests that CSP-6 dephosphorylates WC-1 to a significant degree, and yet the clock still functions with no period lengthening (Fig 1). WC-2 ChIP analysis in the Δ*csp-*6 deletion mutant revealed moderately (1.5–2 fold) reduced WC-2 binding occupancy at the *frq* promoter C-box site as compared to wild type but no loss of rhythm (S9 Fig). The reduced binding is roughly consistent with previous work correlating hyperphosphorylated WCC with reduced DNA binding activity at *frq* C-box promoter [10,13]; however, the WC-1 hyperphosphorylation plainly has little effect on the core clock feedback loop. Previous reports had suggested that transcriptionally active hypophosphorylated WCCs are unstable and that active WCC leads to very low WCC levels [13,16,24]; however, here we saw the reverse, where hyperphosphorylated WCC present in Δ*csp-*6 is much less stable (Fig 2B) but still binds to DNA and supports a robust clock at least for the first few days though not perfectly as that in the wild type. Additionally, we noticed that the FRQ protein level and *frq* mRNA expression level were reduced after moving to constant darkness suggesting that CSP-6 affected the expression of FRQ though not enough to disrupt the clock oscillation (S1B and S1C Fig). Different from other phosphatase proteins so far examined such as PP1, PP2A, PPP-1 or PP4, CSP-6 did not regulate the phosphorylation level of FRQ. Furthermore, we also examined WCC binding to the c-box region over two days. The results showed reduced binding at *frq* C-box in Δ*csp-*6 compared to the wild type (S8B and S8C Fig), so most likely CSP-6 plays a role in maintaining robust FRQ expression through aiding WC-1 binding activity [18,46]. It should be noted, however, that the hyperphosphorylated WC-1 seen in Δ*csp*-6 still binds to the *frq* promoter and still drives a circadian clock.

Loss of CSP-6 resulted in an around 4 hr phase delay in the rhythm, a phenotype similar to that seen strains lacking VVD, the small blue light photoreceptor protein consisting of LOV domain and an N-terminal cap that physically interacts with WC-1 to reduce its ability to activate transcription to regulate photoadaptation [26,27]. Indeed, an additive phase delay was observed in the double mutant Δ*vvd*, Δ*csp-*6 (Fig 4B), and there was plainly much less WC-1 in Δ*vvd*, Δ*csp-*6 compared to either single mutant, suggesting that the low level of WC-1 seen in Δ*csp-*6 might be the reason for the phase delay. However, elevated expression of WC-1 in *wc*-1::*qa*-*wc*-1, Δ*csp-*6 only partially rescued the phase defect (Fig 4D), and this suggested that the reduced VVD-WC-1 interaction also observed in the Δ*csp-*6 mutant, independent of the low level of WC-1, might also underlie the phase delay. This seems to be the case, because even the addition of three times more WC-1 in immunoprecipitations from Δ*csp-*6 (to make up for the reduced WC-1 level in this strain) failed to recover a full level of interaction (Fig 4C, S4A Fig). Therefore we hypothesize that hyperphosphorylation of WC-1 might independently impact the interaction between VVD and WC-1 resulting in the phase delay and weaker light response phenotypes.

WC-1 and WC-2 play multiple roles in the circadian system; their protein levels contribute to the robustness and stability of the clock and they are at the top of the hierarchy of transcription factors that governs circadian output. The importance of the WCC to output was demonstrated by Cheng et al. (2001) who examined the strains *qa*-WC-1 or *qa*-WC-2 in which ORFs of WC-1 or WC-2 were under the control of quinic acid-inducible promoter (*qa*-2); these strains were arrhythmic on race tubes when the QA concentration was less than 1X10^-7^M (corresponding to << 10% of wt WC-1 levels), but the conidiation rhythm became overt and robust as inducer was increased to yield even 30% of normal levels. Our initial observation of less WC-1 in the Δ*csp*-6 mutant suggested that this was the cause of the loss of the overt rhythm; however, we were unable to find evidence for limiting WC-1. Westerns (Fig 6E) showed ADV-1 levels in Δ*csp*-6 comparable to wt and light-induction of WCC target genes was only slightly reduced, not severely reduced as is seen when WC-1 becomes truly limiting as in the *wc*-1[MK1] allele (56). When even full induction of WC-1 in *qa*-*wc*-1 failed to rescue the conidiation rhythmicity (S4B and S4C Fig) a role for CSP-6 specifically in output was suggested. This was supported by finding that the core oscillator runs with a normal period length in the Δ*csp-*6 mutant, although the amplitude of the daily cycle in WCC binding to *frq*, the positive arm in the cycle was reduced compared to wide type (S8B and S8C Fig). Precedents exist for target gene-specific effects of post-translational modifications of transcription factors, for instance in mutants defective in phosphorylation of Ser(276) of NF-kB subunit p65 [47,48].

Circadian output is a consequence of the negative feedback of the central clock that results in the daily cycle of WCC activity. Output happens when the WCC drives expression of *clock-controlled gene*s (*ccg*s) whose products do not impact the core oscillator itself [30,49]. A number of *ccg*s have been identified as involved in circadian output regulation [50–53] including genes encoding transcription factors (TFs) directly controlled by WCC involved in clock and light regulation. From these data emerged the model where WCC sits atop an interconnected hierarchy of TFs that governs light and clock regulation [22]. The results of our study indicate that the transcriptional activity of some TFs within this hierarchy that are required for conidiation rhythms and are downstream of CSP-6-WC-1 were disrupted by deletion of *csp-*6. Examination of candidate TF genes including *csp-*1, *fluffy*, *msn-*1, and *adv-*1 revealed that both transcriptional and translational rhythmicity of *adv-1*/ADV-1 was significantly disrupted in the Δ*csp-*6 mutant (Fig 6C and 6E, S7 Fig). We confirmed previous results showing arrhythmicity of Δ*adv-*1 by race tube assay though with normal core clock function [22] as well as rhythmic WCC binding at the promoter of *adv-*1 [32] that we here show is weakened in Δ*csp-*6 (Fig 6D). These data lead to the surprising conclusion that the phosphorylation status of WC-1 differentially affects its two functions: Loss of CSP-6-mediated dephosphorylation has little impact on WC-1 action in the core circadian oscillator but it abrogates the ability of WCC to regulate a salient circadian output, the daily cycle of conidiation. We also noticed that though the rhythmic expression of *csp*-1 and *fluffy* promoter was not affected by deletion of *csp*-6, the mRNA expression level was reduced for both genes in Δ*csp-*6 (S7E Fig), further suggesting that CSP-6 mediated dephosphorylation of WC-1 has distinct role in output through ADV-1, but also generally affects the expression of other downstream targets.

**Fig 8:**
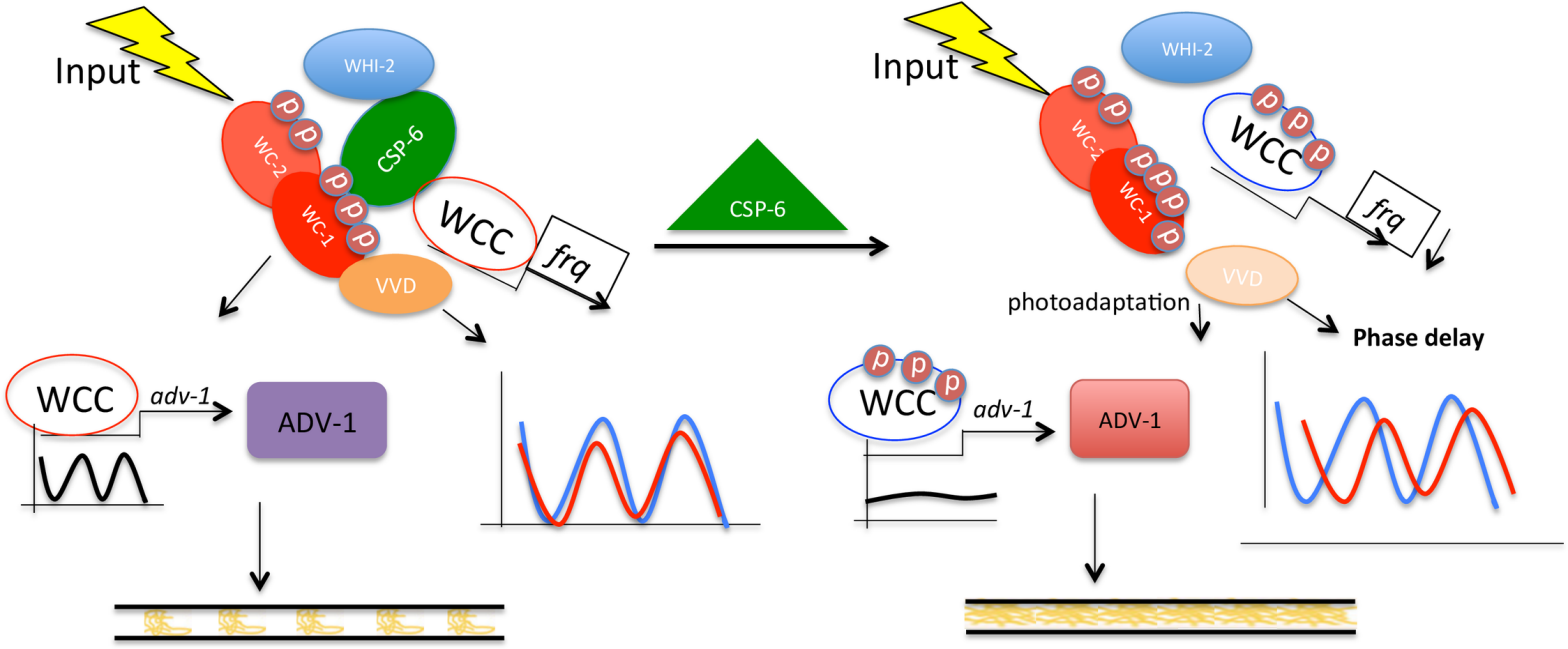
A working model: CSP-6 functions upstream of WC-1-ADV-1 in regulation of the circadian output pathway leading to control of development. (Left) CSP-6 physically interacts with and dephosphorylates WC-1 to an optimal level facilitating its interaction with VVD, regulation of photoadaptation, and circadian phase. Meanwhile, the promoter of *adv-*1 is rhythmically recruited by WC-1 to active the rhythmic transcription and translation of *adv-*1/ADV-1 that is essential for overt rhythms. WHI-2 directly interacts with and assists CSP-6 fully to dephosphorylate WC-1 but does not directly interact with WC-1. (Right) Deletion of CSP-6 results in hyperphosphorylated WC-1 and reduced amounts of WC -1 protein, weakening the interaction between WC-1 and VVD and resulting in a 3.5-hour delayed phase. Furthermore, the hyperphosphorylated WC-1 in Δ*csp*-6 results in disrupted rhythmic binding of WCC to the *adv*-1 promoter and to arrhythmic expression of *adv*-1*/*ADV-1 and resulting in loss of overt circadian output. CSP-6 functions upstream of WC-1/ADV-1 and regulates through WC-1 the expression of *adv-*1/ADV-1.

Based on these data, a working model is summarized in Fig 8. CSP-6 physically interacts with and dephosphorylates WC-1 *in vivo* so that it can interact with VVD to regulate photoadaptation and phase resetting. WHI-2, as an assisting protein, associates with CSP-6 to adjust the WC-1 protein amount and phosphorylation level. *adv*-1, as a *ccg*, is one of the direct targets of the WCC that regulates circadian output. The promoter of *adv-*1 is rhythmically recruited by WCC, and ADV-1 directly functions downstream of CSP-6/WHI-2/WC-1 to control overt rhythmic conidiation on race tubes. In contrast, hyperphosphorylated WC-1 seen in the Δ*csp-6* deletion mutant shows disrupted rhythmic DNA binding activity at the *adv*-1 promoter and arrhythmic transcription/translation of *adv-*1/ADV-1, the essential cause of the circadian output defect in the Δ*csp-*6 mutant. However, hyperphosphorylated WCC in Δ*csp-6* still sufficiently drives rhythmic *frq* expression for the clock to run, though with reduced FRQ levels indicating that CSP-6 plays a role in maintaining robust *frq*/FRQ expression.

These data present some unexpected paradoxes and compel a more nuanced view of the role of phosphorylation of WC-1 in the core clock and in output. The existing model of the circadian feedback loop posits that WC-1 is active and unstable early in the circadian cycle prior to its undergoing changes in phosphorylation that cause it to bind less tightly to DNA. The feedback loop closes when, because of these alterations in the phosphorylation state, WC-1 becomes stable and inactive. It is plausible that the differing combinations of phosphorylation states seen at times throughout the circadian cycle could differentially affect sequence-specific binding affinities of WC-1 to target genes, thereby modulating amplitudes in a hierarchical manner such that some genes are only modestly affected whereas others are more severely affected. Hence, we cannot rule out that the effects on output–specifically the loss of the overt developmental rhythm–arising from loss of CSP-6 are the result of the reduced amount of WC-1 seen in this strain. However, this seems less likely because the reduction in the absolute levels of both WC-1 (including all of it isoforms, Figs 2A and 4A) and ADV-1 (Fig 6E, 6F and 6G), each on the order of three-fold, are not so severe. Instead it seems that it is the quality of WC-1 and not its quantity that is important. Implicit in this statement is the prediction of different classes of phosphosites in WC-1, some involved more directly in oscillator function and some more in output so that output, more than the oscillator itself, is modulated based on the ability of CSP-6 to dephosphorylate WC-1.

CSP-6 is plainly required to dephosphorylate WC-1 because WC-1 is hyperphosphorylated in Δ*csp*-6. The existing model of the circadian feedback loop posits that WC-1 is active and unstable early in the circadian cycle when it is hypophosphorylated, and the feedback loop closes when, because of hyperphosphorylation, WC-1 becomes stable and inactive. Here, paradoxically, WC-1 is hyperphosphorylated, active, and unstable, and the clock runs, indicating that the class of phosphosites normally dephosphorylated by CSP-6 is distinct from the phosphosites mediating closure of the feedback loop. Different combinatorial states of WC-1 phosphorylation affecting DNA affinity at different promoters, along with turnover, replenishment, and inactivation, are all likely to contribute to distinguishing between oscillator and output functions of WC-1.

## Materials and methods

### Strains and culture conditions

The *ras*-1^bd^ and 74A strains were used as clock WT strains in this study. The Δ*csp-*6, Δ*psr-*2, *Δwhi-*2, Δ*adv*-1, Δ*vvd* strains were obtained from the Fungal Genetics Stock Center [41]. These KO strains were backcrossed to *ras*-1^bd^ to obtain band phenotype for race tube assays. The newly created double knock-out strains were Δ*csp-*6, Δ*psr-*2 and Δ*csp-*6, Δ*whi-*2 in background #1497 (*mus*52::*natamycin*). *Neurospora* transformation was done as previously described [41].

Race tube medium contained 1xVogel’s salts, 0.1% glucose, 0.17% arginine, 50ng/mL biotin and 1.5% (w/v) agar. Race tube assays were carried out as previously described [54]. Liquid cultures were grown in medium containing 1xVogel’s, 0.5% arginine, and 50ng/mL biotin with 2% glucose.

### Protein isolation and detection

Protein extraction, quantification, Western blot analysis and Co-IP were performed as described previously [55,56]. For Western blot analysis, equal amounts of total proteins (30 μg) were loaded to protein gels that were transferred to PVDF membrane after electrophoresis. The V5 antibody (Invitrogen, NY) was used at dilution of 1:5000. Other antibodies including antisera directed at WC-1, WC-2, and FRQ were generated by our own lab [57]. For Co-IP, 2mg total protein was incubated with 30 μl V5 agarose beads (Sigma, MO) for 2h to overnight at 4°C in PEB buffer (50mM HEPES, pH7.4, 150mM NaCl, 10% glycerol, 0.4% NP-40). The V5 agarose beads were washed with wash buffer (50mM HEPES, pH7.4, 150mM NaCl, 0.4% NP-40) four times and eluted with 4xLDS buffer (Thermo Fisher, MA) at 95°C for 5min. In testing the interaction between WC-1 and VVD in Δ*csp-*6, because of the reduced WC-1 level detected in the Δ*csp-*6 mutant and to make sure that similar amounts of WC-1 were available based on the quantification by western blot (S4A Fig) we used three fold more input of *vvd*^V5^, Δ*csp*-6 than of *vvd*^V5^, and of the wild type (*ras-*1^bd^). The same treatment was performed for the interaction between WC-1 and ADV-1 with DSP crosslink as described previously [11].

For protein purified by tandem affinity tag, total protein was isolated from 10–15 g of fresh tissue and incubated with FLAG agarose beads first, followed by V5 magnetic beads. A small amount of the final V5 precipitates were separated by SDS-PAGE and the gel was silver-stained followed manufacturer’s instruction for purification quality examination (SilverQuest, Invitrogen). For Mass Spectrometry, the remainder of the V5 precipitate preparation was separated by SDS-PAGE and the specific bands were excised from a Coomassie blue stained gel and sent for Mass Spectrometry as previously reported [44]. Nuclear preparation was performed as reported [58].

### RNA isolation and quantitative RT-PCR

Total RNA was isolated by Trizol according to the manufacturer’s protocol (15596–026; Invitrogen). For quantitative RT-PCR, 2–3 μg RNA were treated with DNase at 37°C for 60min and then incubated with inactivation buffer for 5 min following instructions (AM2239; Life Technology). cDNA was generated by reverse transcription reaction according to the manufacturer’s protocol (18080–051; Invitrogen). Expression levels of genes of interest were analyzed by quantitative real-time PCR with primers listed in S1 Table in the Supplemental Material.

### Luciferase reporter assay

The luciferase reporter assay was performed as described previously [36]. The C-box*-luc*, csr::C-box*-luc*, *ras*-1^bd^, A or *his*-3::*pfrq-luc*, *ras*-1^bd^, *A*/*a* were used as control strains. Knock out strains were crossed with these as appropriate to place *frq-luc* at the *csr* or *his*-3 locus. Camera runs showed there was no difference in rhythmicity of the *frq-luc* reporter at *csr* versus the *his-*3 locus. Race tube medium was used for luciferase assays and 0.01M quinic acid (QA) was added for *qa-*2 promoter-driven strains as appropriate. All cultures were grown in LL for 2 days and then transferred to constant darkness and luminescence was recorded every hour for six days.

### ChIP and region-specific ChIP PCR

ChIP assays were performed as described previously [22,59]. Briefly, the *Neurospora* tissues were fixed with 1% formaldehyde for 15 min and quenched by glycine at final concentration of 125mM for 5 min. Around fifty-mg of cross-linked tissue was used for each sample and were suspended in 500 μl ChIP lysis buffer. Chromatin was sheared by sonication to 100–500 bp fragments. The immunoprecipitation was performed using 5μl WC-2 antibody [57]. Immunoprecipitated DNA was quantified using real time PCR with primer sets listed in S1 Table. ChIP quantitative PCR data were normalized to a sample of input DNA as described in instructions from the Life Technology website (https://www.lifetechnologies.com/us/en/home/life-science/epigenetics-noncoding-rna-research/chromatin-remodeling/chromatin-immunoprecipitation-chip/chip-analysis.html). Each experiment was independently performed at least three times.

## Supporting information

S1 Fig CSP-6 is not a typically rhythmic circadian protein and is important for maintaining robust FRQ expression. A: Schematic depiction of the domain architecture of CSP-6 protein based on NCBI BLAST. B. Western blot analysis showing FRQ remains rhythmicity but with low protein levels in Δ*csp-6*. Cultures were harvested in constant darkness at the indicated times during the second day in darkness.
C: *frq* mRNA accumulates with a circadian rhythm in the Δ*csp-6* mutant but with reduced amplitude compared to wild type in the darkness from 24-48hrs. *frq* mRNA expression was assayed by qRT-PCR and normalized to *rac-*1 in *ras*-1^bd^ and *ras*-1^bd^,Δ*csp-*6. The rhythm in Δ*csp-6* mutant is weaker than that in the wild type and the phase is around 4-hour delay. D: Luciferase activity of *frq C-box*-*luc* and the *csp-6* promoter driving luciferase under free running conditions shows that *csp*-6 is only very weakly circadianly regulated. E: Luciferase activity of *frq C-box*-*luc* and the CSP-6 translational luciferase showing CSP-6 is not a rhythmically expressed protein. F: Western blot analysis showing a time course (LL-DD48h) of CSP-6 protein expression at 4 h resolution; hours after light to dark transfer are shown above the blots and the densitometric analysis of these Western blot data are shown in the left panel.
(TIF)

S2 Fig A CSP-6 paralog PSR-2 encoded by NCU08948 is not important for the circadian clock or circadian output in *Neurospora crassa*. A: Amino acid sequence alignment of CSP-6 and its paralog PSR-2 (NCU08948) showing they are conserved within the C-terminal phosphatase domain. Protein sequence alignment was performed by EBI-cluster. Four amino acids DLDE in red frame depicting the conservation of the active site motif in both HAD phosphatase proteins, CSP-6 and PSR-2. B: Race tube assays of WT (*ras-*1^bd^) and Δ*psr*-2, *ras*-1^bd^. Normal conidiation rhythms were observed in strains lacking *psr*-2, though with slight growth defect; duplicate race tubes are shown for each strain. Period is reported in hours ± one standard deviation C: Luciferase traces of *frq-luc* in *ras-*1^bd^ and Δ*psr-*2, *ras-1*^bd^.
(TIF)

S3 Fig A carotenoid biosynthesis defect is observed in Δ*csp-6*. A. Western blot analysis showing WC-1 in WT, Δ*csp-6* and *csp-*6^ΔDLDE^ with or without the λ-phosphatase treatment under indicated conditions. DD24: constant darkness for 24hr; LP30: light pulse for 30min. B: Strains of *ras-*1^bd^; Δ*csp-*6, *ras*-1^bd^; Δ*vvd*, *ras*-1^bd^, and the double mutant Δ*csp*-6, Δ*vvd*, *ras-*1^bd^ were grown on minimal slants showing their carotenoid accumulation and growth defect. C: Strains (*ras*-1^bd^ and Δ*csp-*6, *ras*-1^bd^) exposed to a 30 min light pulse (LP30) were subjected to RT-PCR to determine mRNA expression levels of three *albino* genes (*al*-1, *al-*2, *al-*3) as a measure of impaired light responses and carotenoid biosynthesis. D: Real time PCR analysis of light inducible genes (*al-*1, *al-*3, *sub*-1, *frq*) in strains *ras*-1^bd^ and Δ*csp-*6, *ras*-1^bd^ with light pulse samples. E: Western blot analysis showing reduced amounts of WC-2 protein but no significant effect on WC-2 phosphorylation in Δ*csp*-6.
(TIF)

S4 Fig Increasing WC-1 expression in the Δ*csp-6* background fails to rescue overt rhythmicity on race tube. A: Western blot analysis showing approximately three times more WC-1 protein in *ras*-1^bd^ as in Δ*csp*-6, *ras-*1^bd^. B: Western blot showing WC-1 protein expression in WT and in a *qa-*2*-*driven *wc-*1 strain at the native locus in Δ*csp-*6. In the presence of 10^−2^ M QA, WC-1 levels in the *qa-*2 driven *wc*-1 strain in Δ*csp*-6 were similar to those in wild type. The WC-1 was still hyperphosphorylated in Δ*csp-*6, and a dephosphorylation assay with λPPase showed the lower mobility of WC-1 in Δ*csp-*6 was caused by phosphorylation. The protein level of WC-1 was not affected by exogenous QA in the clock wild type strain. The unspecific band was used to validate the quantity of protein loading. C: Luciferase traces for three technical replicates of *frq-luc* in *wc*-1::*qa*:*wc*-1 with 10^-2^M QA; the only source of WC-1 in this strain is the QA-induced construct. D: Race tube assay showing that even in the presence of QA to elevate WC-1 expression, no conidiation banding was observed in the *wc*-1::*qa*:*wc-*1, Δ*csp*-6 strain while *ras-*1^bd^ showed rhythmic banding on race tube with 10^-2^M QA.
(TIF)

S5 Fig CSP-6 has different isoforms and translational post-modifications. A: Two translational start sites, labeled as S1 and S2, were found based on CSP-6 sequence as reported by FungiDB [http://fungidb.org/fungidb/]; the difference in size between the two translational isoforms was 5.5kDa. B: Western blots showing the two isoforms of CSP-6 and their modification. Isoform S1 is obviously phosphorylated, and isoform S2 likely phosphorylated, based on results from phosphatase treatment. Buffer: protein extraction buffer; PP: phosphatase; PPI: phosphatase inhibitor.
(TIF)

S6 Fig WHI-2 does not contribute substantially to WC-1 phosphorylation. A: WC-1 is hyperphosphorylated in the Δ*whi*-2. Shown is a Western blot of WC-1 in *ras-*1^bd^ and Δ*whi*-2, *ras*-1^bd^, the conditions are label as indicated on the top of the western. B: Western blot analysis showing no difference in WC-1 protein amount, or in degree of hyperphosphorylation, between Δ*csp-6* and the double mutant Δ*csp-*6, Δ*whi-*2.
(TIF)

S7 Fig Screening of WC-1 downstream targets for roles for CSP-6. A: Race tube assay showing Δ*msn*-1 displays normal overt rhythmic banding but a significantly reduced growth rate; triplicate race tubes are shown. B: Luciferase traces elucidating that a functional clock was running in the Δ*msn*-1 mutant; duplicate assays are shown. C-D: Representative luciferase activity assays showing circadian rhythmicity of *csp*-1 (C) and *fluffy* (D) promoter activity was not abolished in the Δ*csp*-6 mutant. E: Strains *ras*-1^bd^ and Δ*csp-*6, *ras*-1^bd^ were subjected to RT-PCR to determine mRNA expression levels of *csp*-1 and *fluffy* normalize to WT (*ras*-1^bd^), error bars represent +/- S.D.
(TIF)

S8 Fig Chip analysis showing WCCs binding efficiency was reduced in Δ*csp-6*. A: ChIP analysis showing the recruitment of WC-2 to the *adv*-1 promoter in *ras*-1^bd^ and *ras-*1^bd^, Δ*csp-*6 under indicated conditions. LL: constant light for 24h, LP15’: light pulse for 15min after moving from DD24. B. C: Chip analysis showing the recruitment of WC-2 to the *frq* promoter C-box in Δ*csp-*6 is reduced compared to WT but is still rhythmic with the same peak phase. The examined time points are in darkness from 8h-24h (B) and 28-48h (C). Error bars represent +/- S.D.
(TIF)

S9 Fig CSP-6 does not interact with ADV-1. A: Co-IP assay demonstrating that ADV-1 failed to interact with CSP-6 even using DSP crosslink. ADV-1 was purified by V5 agarose beads from strain *adv*-1^V5^, *csp-*6^FLAG^and no CSP-6 was detected in IP sample. B: Western blot analysis showing there was no change in the phosphorylation status of ADV-1 between WT and Δ*csp-*6 under the indicated conditions. C: A phosphorylation assay showed there was no difference of ADV-1 before and after treatment in wild type and Δ*csp-*6 under the indicated conditions.
(TIF)

S10 Fig Protein sequence alignment of CSP-6 in *Neurospora* and its paralog Psr1p in yeast. Amino acid PKKKKG in red frame indicates a putative nuclear localization site in CSP-6.
(TIF)

S1 Table Primer sets used for quantitative PCR. (DOCX)

S2 Table Listed of CSP-6 interactome identified by MS/MS from sliced gels of purified CSP-6. (DOCX)
