# Supplementary figures and images for: A HAD family phosphatase CSP-6 regulates the circadian output pathway in Neurospora crassa
Source: PLoS Genet. 2018 Jan 19;14(1):e1007192. doi: 10.1371/journal.pgen.1007192 (PMC5800702; doi:10.1371/journal.pgen.1007192)

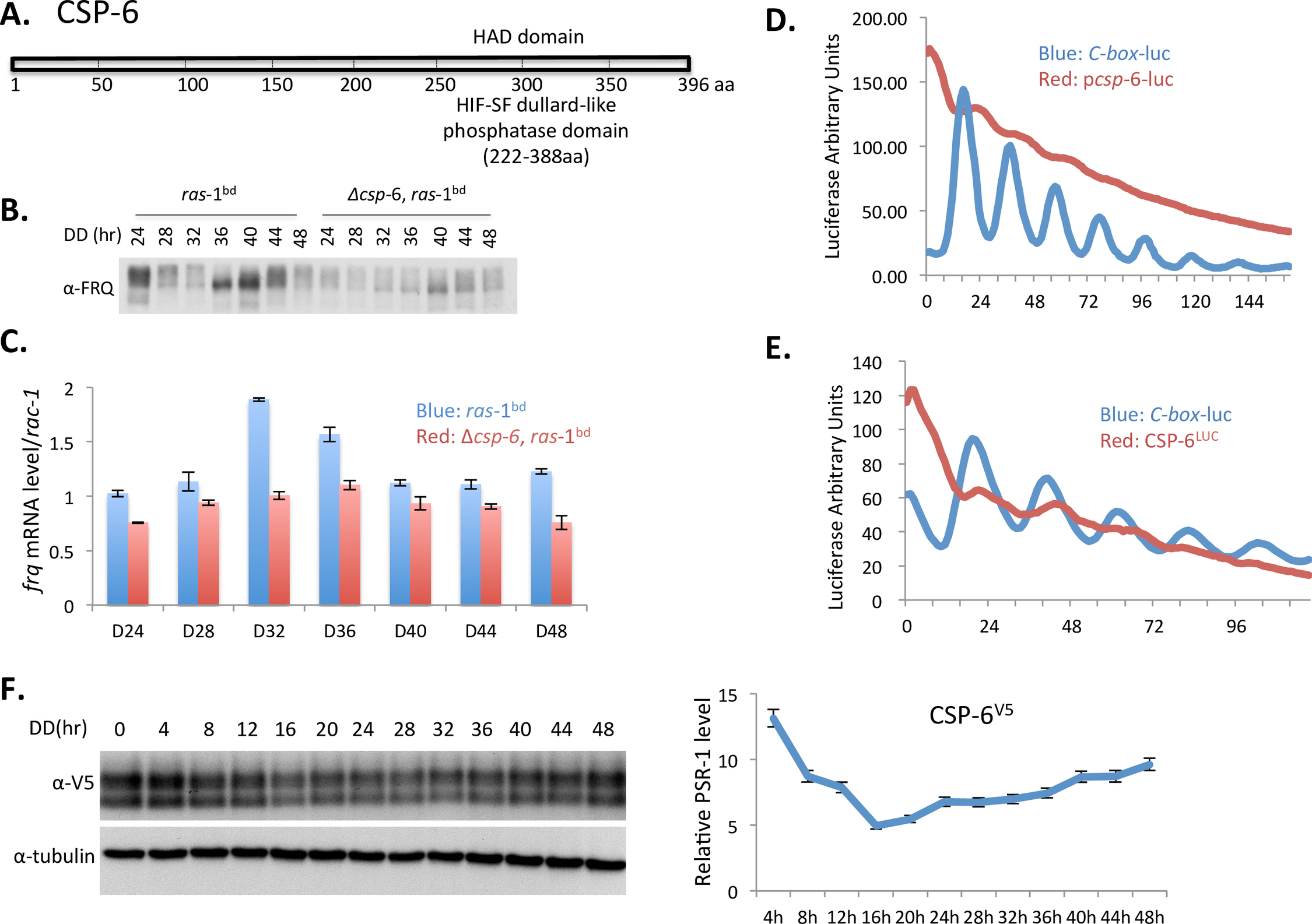

Supplement: S1 Fig — A: Schematic depiction of the domain architecture of CSP-6 protein based on NCBI BLAST. B. Western blot analysis showing FRQ remains rhythmicity but with low protein levels in Δcsp-6. Cultures were harvested in constant darkness at the indicated times during the second day in darkness. C: frq mRNA accumulates with a circadian rhythm in the Δcsp-6 mutant but with reduced amplitude compared to wild type in the darkness from 24-48hrs. frq mRNA expression was assayed by qRT-PCR and normalized to rac-1 in ras-1bd and ras-1bd,Δcsp-6. The rhythm in Δcsp-6 mutant is weaker than that in the wild type and the phase is around 4-hour delay. D: Luciferase activity of frq C-box-luc and the csp-6 promoter driving luciferase under free running conditions shows that csp-6 is only very weakly circadianly regulated. E: Luciferase activity of frq C-box-luc and the CSP-6 translational luciferase showing CSP-6 is not a rhythmically expressed protein. F: Western blot analysis showing a time course (LL-DD48h) of CSP-6 protein expression at 4 h resolution; hours after light to dark transfer are shown above the blots and the densitometric analysis of these Western blot data are shown in the left panel. (TIF) [file pgen.1007192.s001.tif]

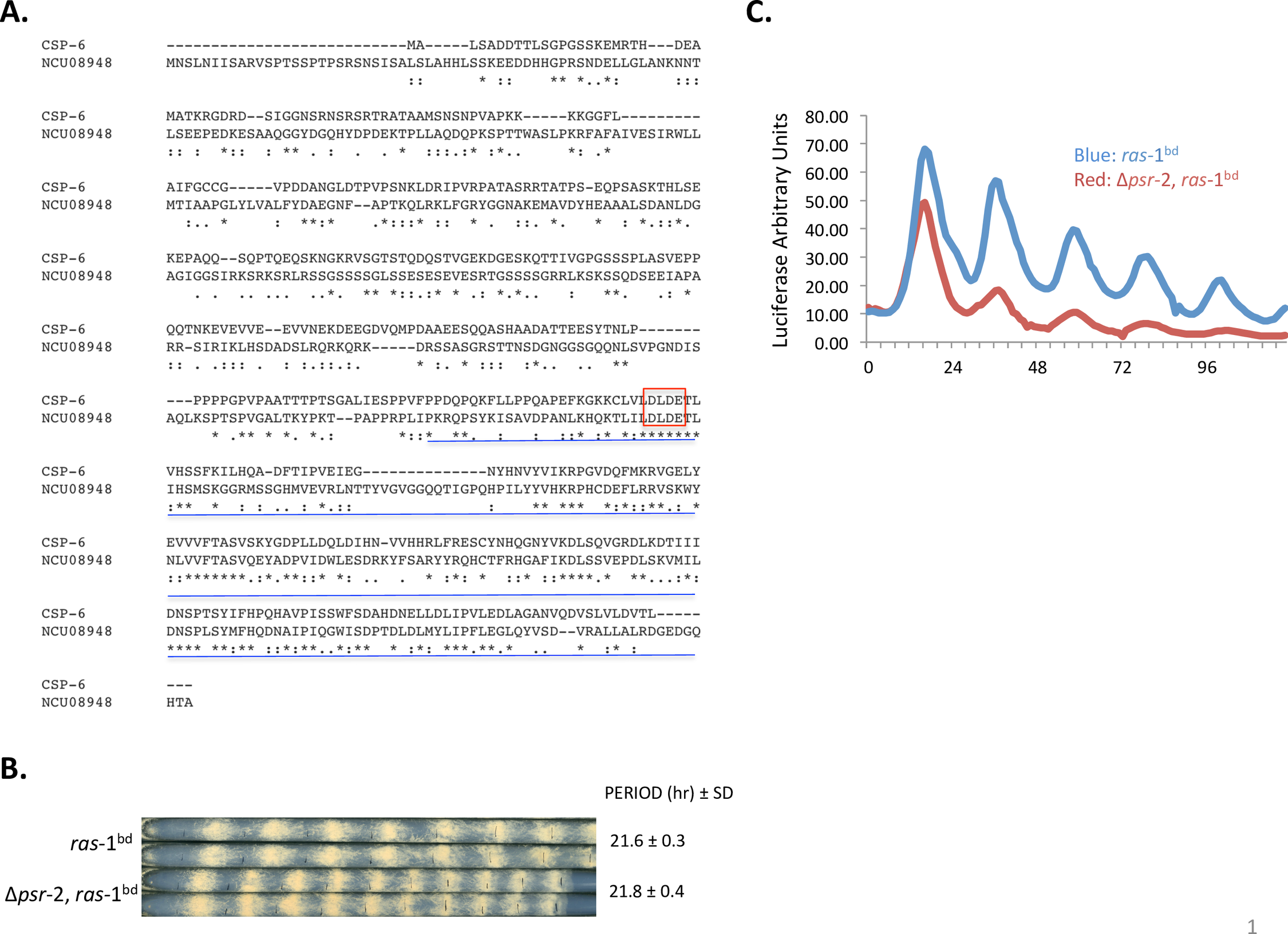

Supplement: S2 Fig — A: Amino acid sequence alignment of CSP-6 and its paralog PSR-2 (NCU08948) showing they are conserved within the C-terminal phosphatase domain. Protein sequence alignment was performed by EBI-cluster. Four amino acids DLDE in red frame depicting the conservation of the active site motif in both HAD phosphatase proteins, CSP-6 and PSR-2. B: Race tube assays of WT (ras-1bd) and Δpsr-2, ras-1bd. Normal conidiation rhythms were observed in strains lacking psr-2, though with slight growth defect; duplicate race tubes are shown for each strain. Period is reported in hours ± one standard deviation C: Luciferase traces of frq-luc in ras-1bd and Δpsr-2, ras-1bd. (TIF) [file pgen.1007192.s002.tif]

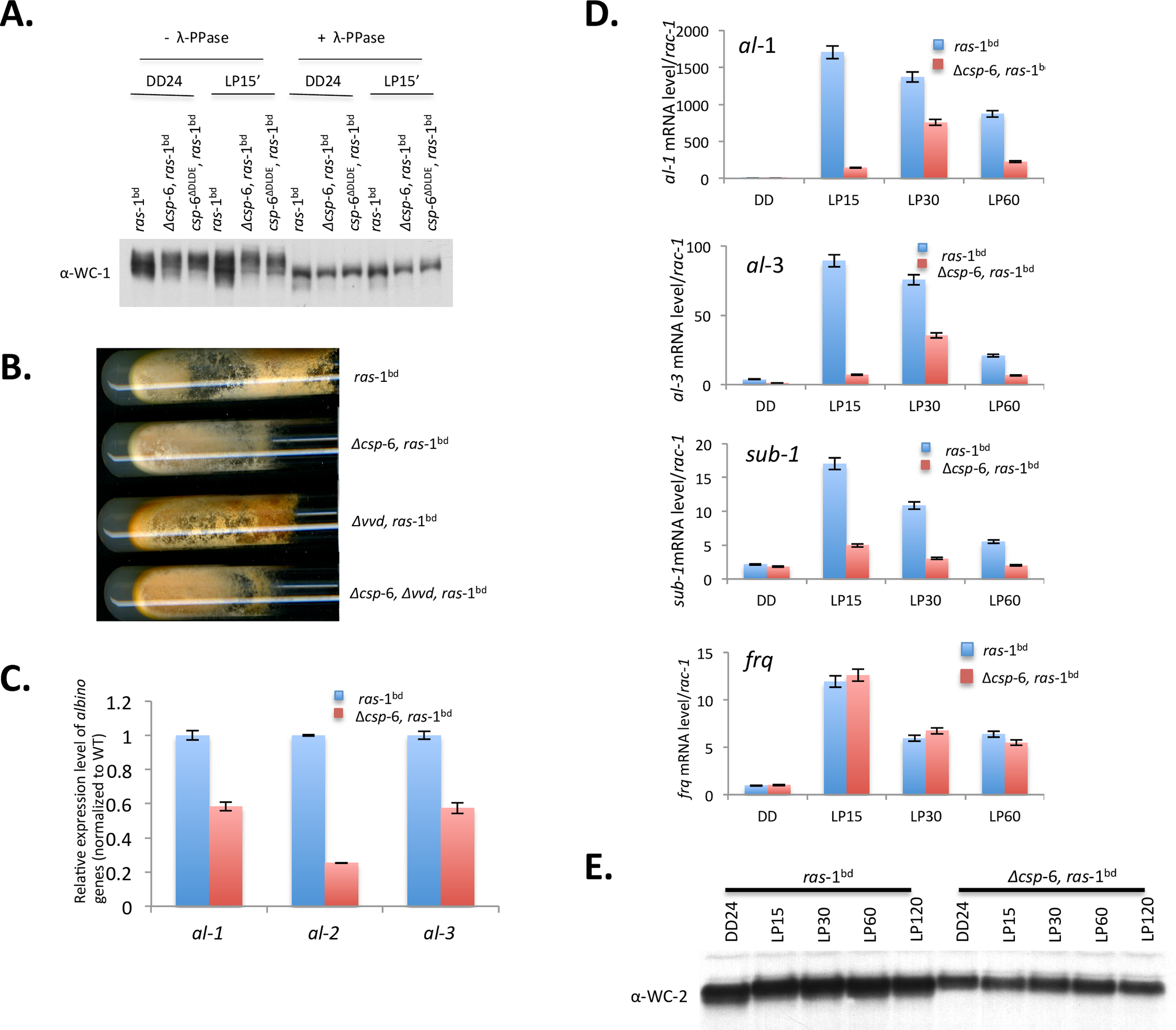

Supplement: S3 Fig — A. Western blot analysis showing WC-1 in WT, Δcsp-6 and csp-6ΔDLDE with or without the λ-phosphatase treatment under indicated conditions. DD24: constant darkness for 24hr; LP30: light pulse for 30min. B: Strains of ras-1bd; Δcsp-6, ras-1bd; Δvvd, ras-1bd, and the double mutant Δcsp-6, Δvvd, ras-1bd were grown on minimal slants showing their carotenoid accumulation and growth defect. C: Strains (ras-1bd and Δcsp-6, ras-1bd) exposed to a 30 min light pulse (LP30) were subjected to RT-PCR to determine mRNA expression levels of three albino genes (al-1, al-2, al-3) as a measure of impaired light responses and carotenoid biosynthesis. D: Real time PCR analysis of light inducible genes (al-1, al-3, sub-1, frq) in strains ras-1bd and Δcsp-6, ras-1bd with light pulse samples. E: Western blot analysis showing reduced amounts of WC-2 protein but no significant effect on WC-2 phosphorylation in Δcsp-6. (TIF) [file pgen.1007192.s003.tif]

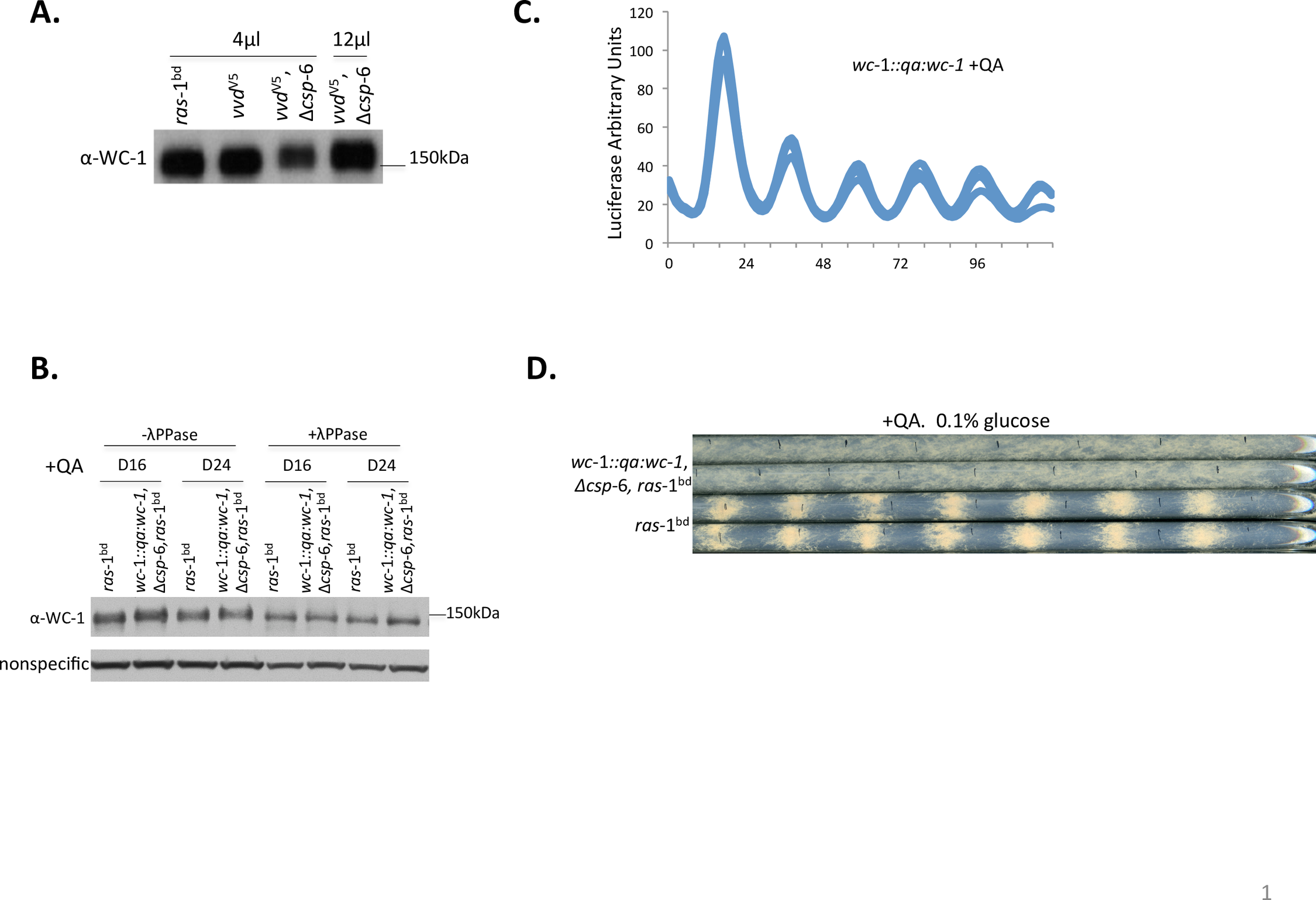

Supplement: S4 Fig — A: Western blot analysis showing approximately three times more WC-1 protein in ras-1bd as in Δcsp-6, ras-1bd. B: Western blot showing WC-1 protein expression in WT and in a qa-2-driven wc-1 strain at the native locus in Δcsp-6. In the presence of 10−2 M QA, WC-1 levels in the qa-2 driven wc-1 strain in Δcsp-6 were similar to those in wild type. The WC-1 was still hyperphosphorylated in Δcsp-6, and a dephosphorylation assay with λPPase showed the lower mobility of WC-1 in Δcsp-6 was caused by phosphorylation. The protein level of WC-1 was not affected by exogenous QA in the clock wild type strain. The unspecific band was used to validate the quantity of protein loading. C: Luciferase traces for three technical replicates of frq-luc in wc-1::qa:wc-1 with 10-2M QA; the only source of WC-1 in this strain is the QA-induced construct. D: Race tube assay showing that even in the presence of QA to elevate WC-1 expression, no conidiation banding was observed in the wc-1::qa:wc-1, Δcsp-6 strain while ras-1bd showed rhythmic banding on race tube with 10-2M QA. (TIF) [file pgen.1007192.s004.tif]

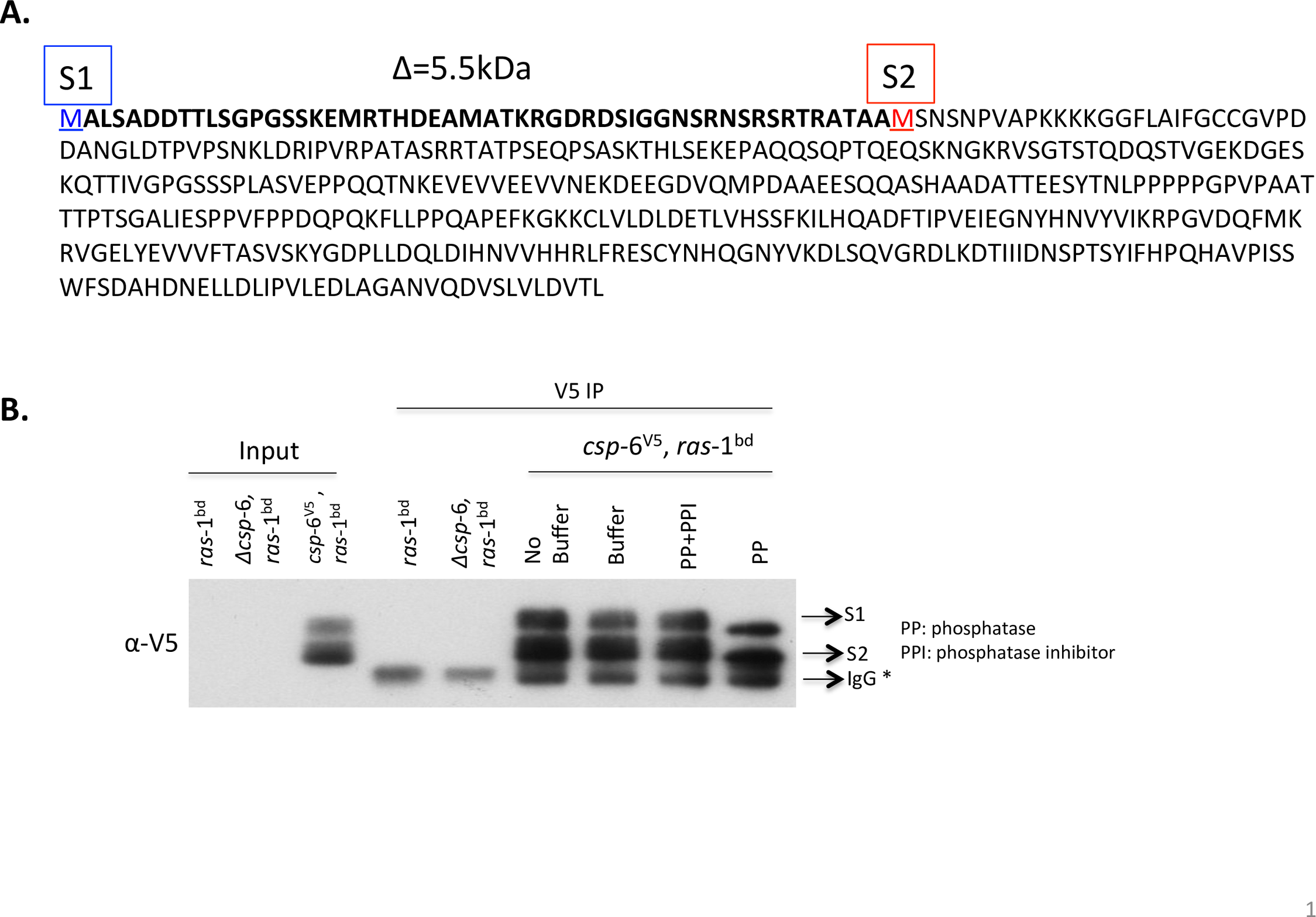

Supplement: S5 Fig — A: Two translational start sites, labeled as S1 and S2, were found based on CSP-6 sequence as reported by FungiDB [http://fungidb.org/fungidb/]; the difference in size between the two translational isoforms was 5.5kDa. B: Western blots showing the two isoforms of CSP-6 and their modification. Isoform S1 is obviously phosphorylated, and isoform S2 likely phosphorylated, based on results from phosphatase treatment. Buffer: protein extraction buffer; PP: phosphatase; PPI: phosphatase inhibitor. (TIF) [file pgen.1007192.s005.tif]

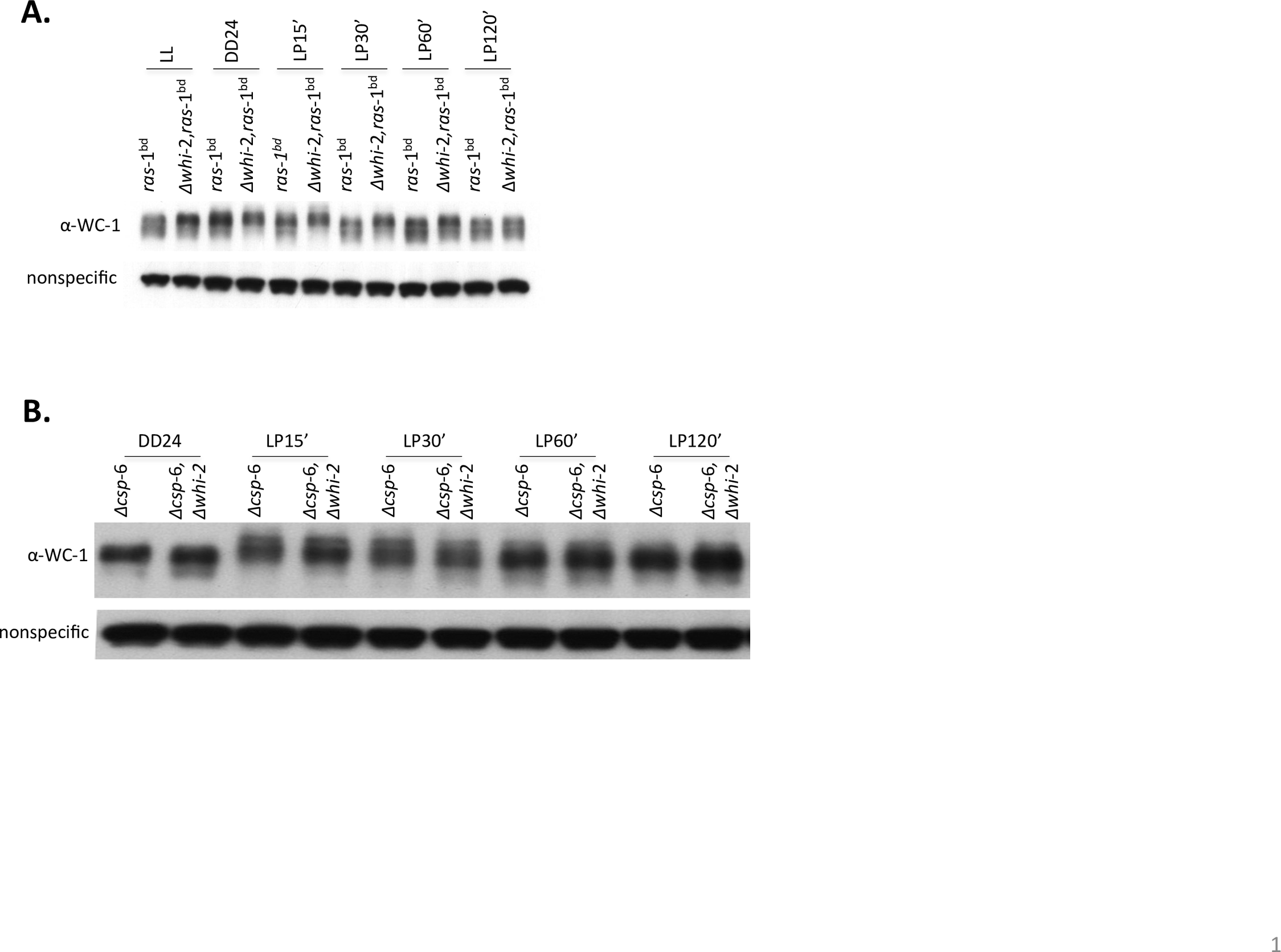

Supplement: S6 Fig — A: WC-1 is hyperphosphorylated in the Δwhi-2. Shown is a Western blot of WC-1 in ras-1bd and Δwhi-2, ras-1bd, the conditions are label as indicated on the top of the western. B: Western blot analysis showing no difference in WC-1 protein amount, or in degree of hyperphosphorylation, between Δcsp-6 and the double mutant Δcsp-6, Δwhi-2. (TIF) [file pgen.1007192.s006.tif]

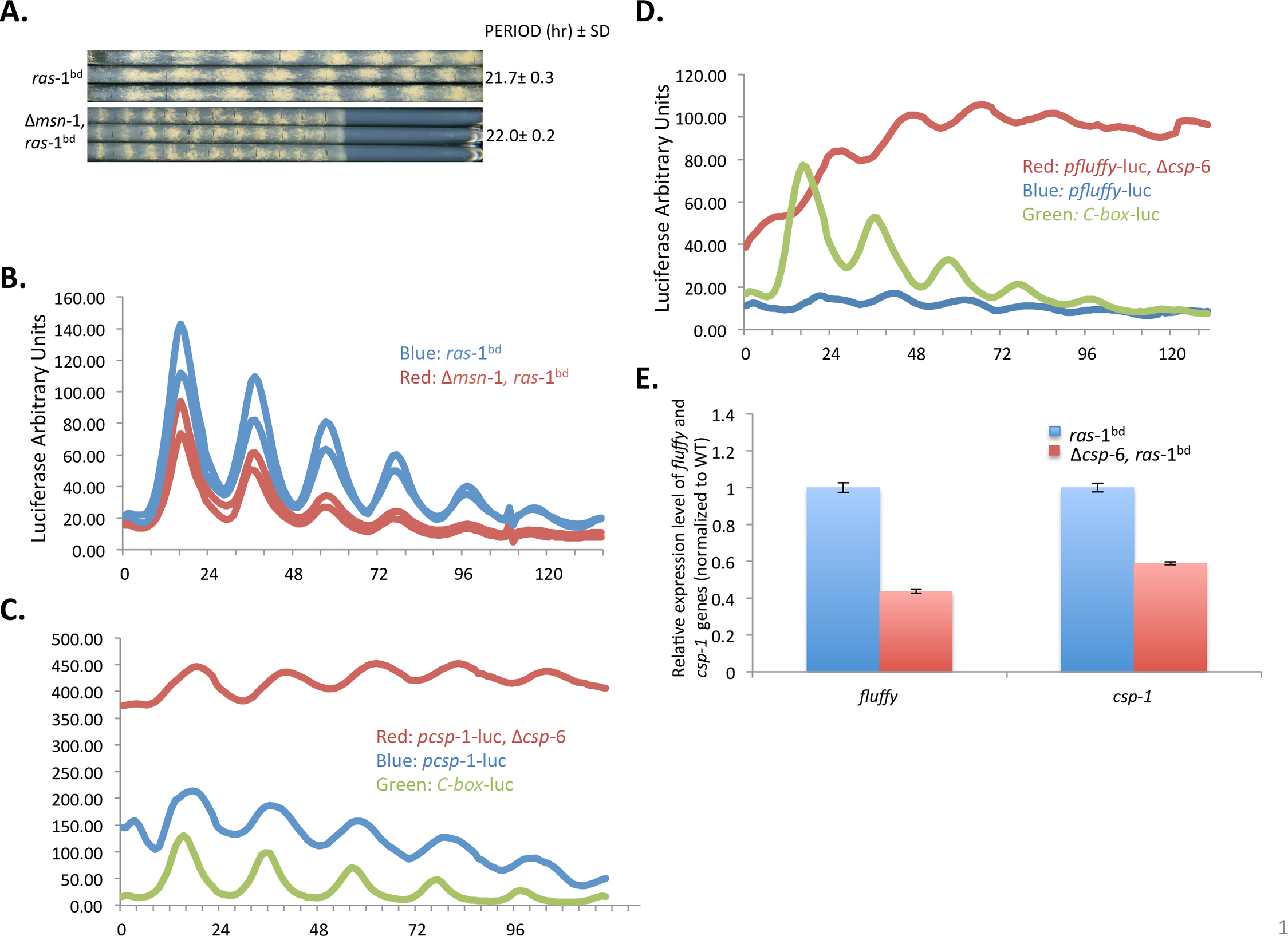

Supplement: S7 Fig — A: Race tube assay showing Δmsn-1 displays normal overt rhythmic banding but a significantly reduced growth rate; triplicate race tubes are shown. B: Luciferase traces elucidating that a functional clock was running in the Δmsn-1 mutant; duplicate assays are shown. C-D: Representative luciferase activity assays showing circadian rhythmicity of csp-1 (C) and fluffy (D) promoter activity was not abolished in the Δcsp-6 mutant. E: Strains ras-1bd and Δcsp-6, ras-1bd were subjected to RT-PCR to determine mRNA expression levels of csp-1 and fluffy normalize to WT (ras-1bd), error bars represent +/- S.D. (TIF) [file pgen.1007192.s007.tif]

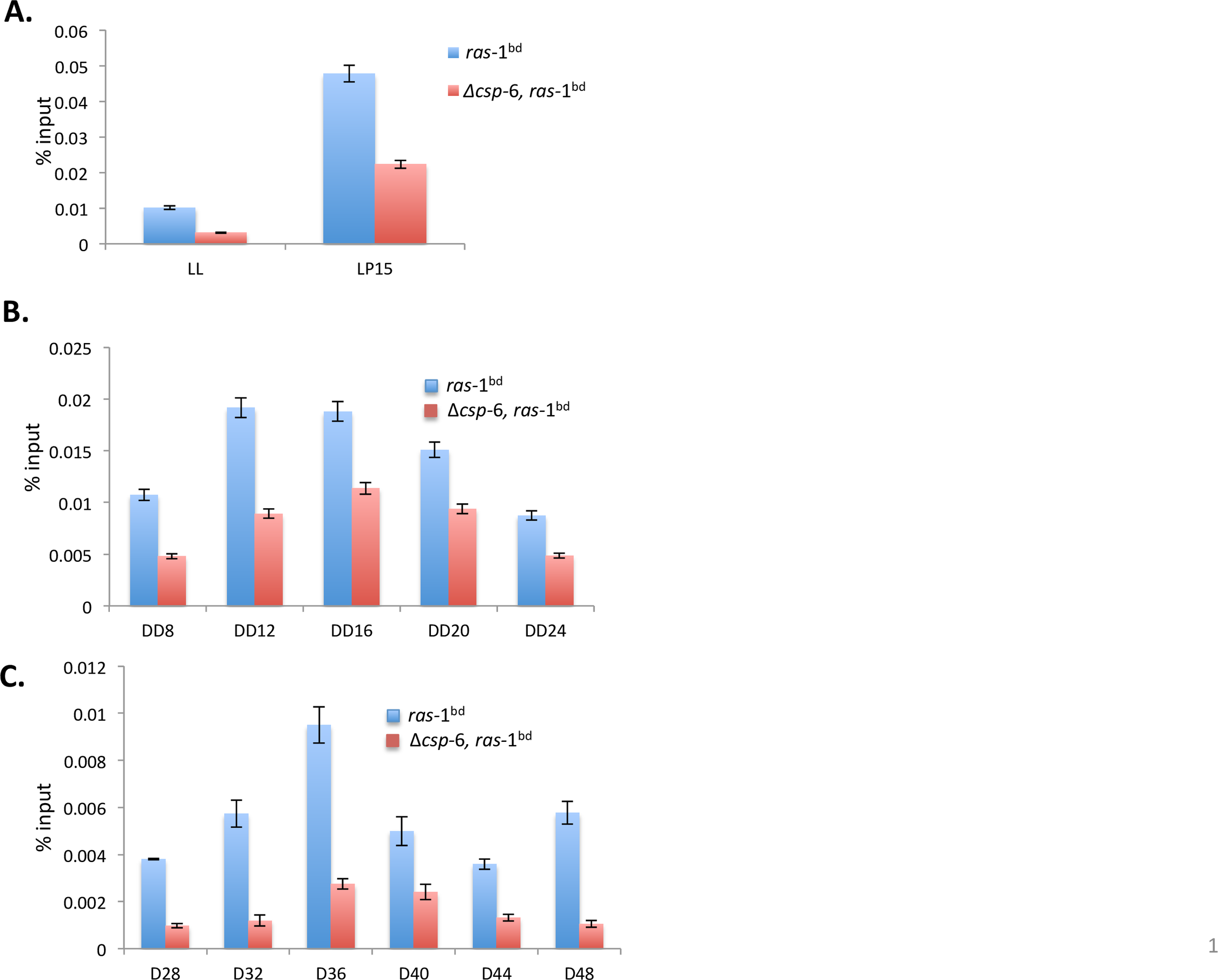

Supplement: S8 Fig — A: ChIP analysis showing the recruitment of WC-2 to the adv-1 promoter in ras-1bd and ras-1bd, Δcsp-6 under indicated conditions. LL: constant light for 24h, LP15’: light pulse for 15min after moving from DD24. B. C: Chip analysis showing the recruitment of WC-2 to the frq promoter C-box in Δcsp-6 is reduced compared to WT but is still rhythmic with the same peak phase. The examined time points are in darkness from 8h-24h (B) and 28-48h (C). Error bars represent +/- S.D. (TIF) [file pgen.1007192.s008.tif]

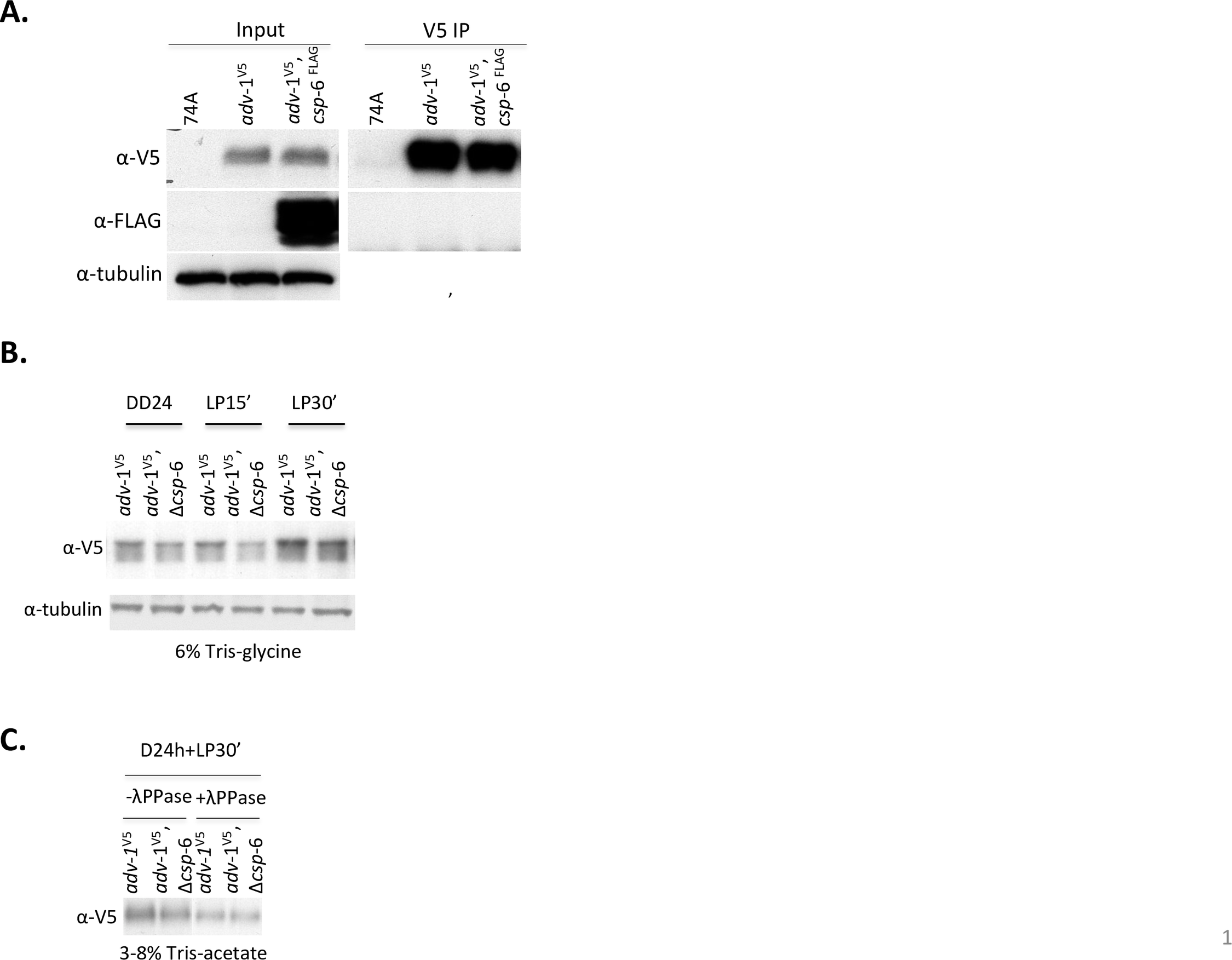

Supplement: S9 Fig — A: Co-IP assay demonstrating that ADV-1 failed to interact with CSP-6 even using DSP crosslink. ADV-1 was purified by V5 agarose beads from strain adv-1V5, csp-6FLAGand no CSP-6 was detected in IP sample. B: Western blot analysis showing there was no change in the phosphorylation status of ADV-1 between WT and Δcsp-6 under the indicated conditions. C: A phosphorylation assay showed there was no difference of ADV-1 before and after treatment in wild type and Δcsp-6 under the indicated conditions. (TIF) [file pgen.1007192.s009.tif]

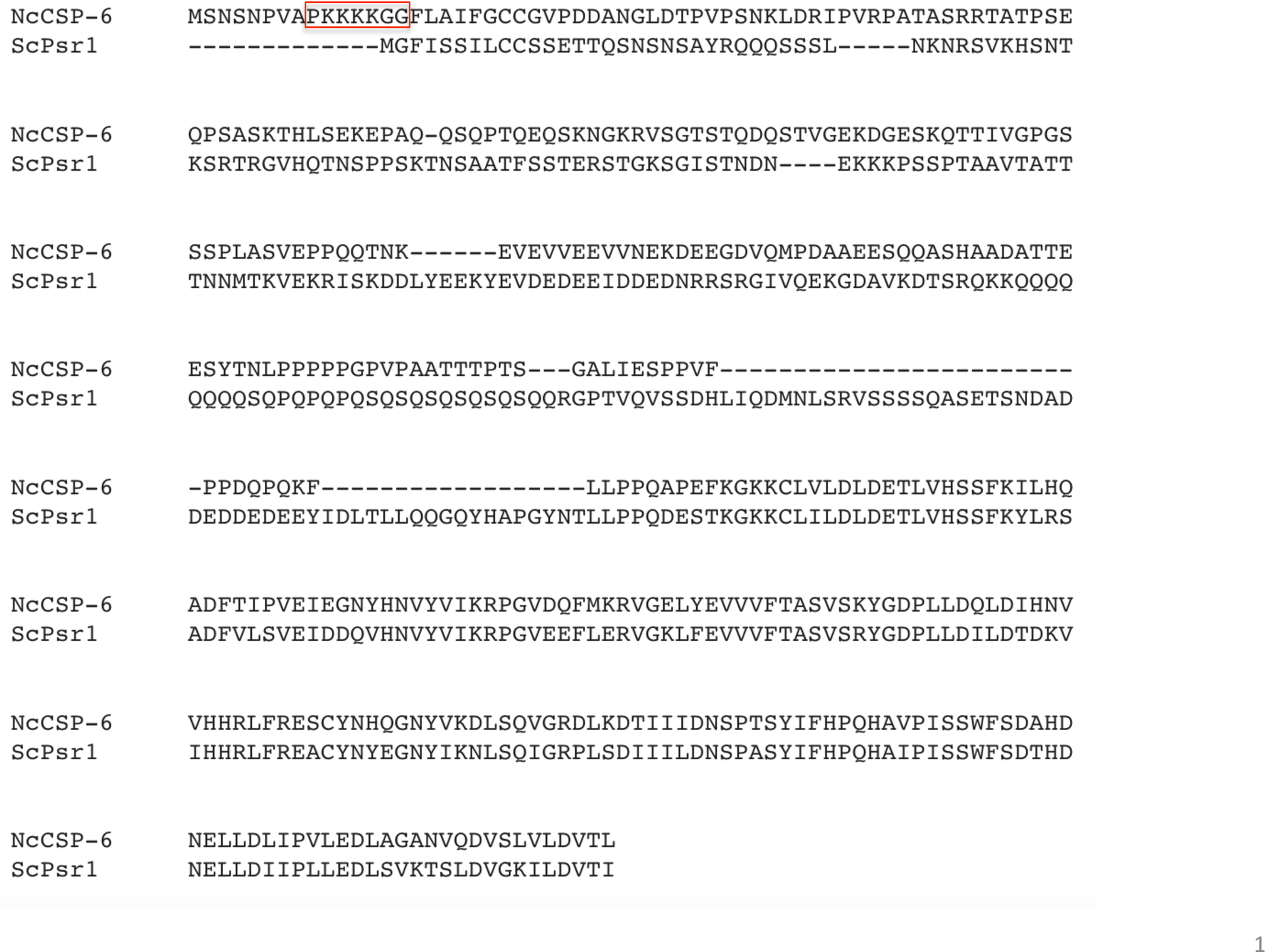

Supplement: S10 Fig — Amino acid PKKKKG in red frame indicates a putative nuclear localization site in CSP-6. (TIF) [file pgen.1007192.s010.tif]
