# Supplementary material for: A HAD family phosphatase CSP-6 regulates the circadian output pathway in Neurospora crassa
Source: PLoS Genet. 2018 Jan 19;14(1):e1007192. doi: 10.1371/journal.pgen.1007192 (PMC5800702; doi:10.1371/journal.pgen.1007192)
Supplement: S1 Table — (DOCX) [file pgen.1007192.s011.docx]

**S1 Table. Primer sets used for quantitative PCR**

| Primer name | Primer sequences |
| --- | --- |
| *frq* qF | TGGCTCGGATAAGAATGGTC |
| *frq* qR | ATGAAAGGTGTCCGAAGGTG |
| *rac-1* qF | GTCCAAGTGGCACCCCGAGAT |
| *rac-1* qR | TCCTTGGCGCAGTTGACACC |
| *wc-1* qF | TCAGCAGCATCAGTTCAACC |
| *wc-1* qR | GTTGATGTTCGCCCATCTCT |
| *al-1* qF | CGCTATCTTTGAGAGGCAGG |
| *al-1* qR | TCTGGTTGGCCGTTTTGG |
| *al-2* qF | TGTACGACTATGCTTTTGTTCA |
| *al-2* qR | AGGAAGCCTGTTTGGATGAG |
| *al-3* qF | TCCTTCTCGCCATACACAATG |
| *al-3* qR | GAACCTTCTCCTTCTCTTCGG |
| *adv-1* qchipF | GCTCAAGATCCCAGTCGATCTCG |
| *adv-1* qchipR | GGTGGTGGACAGTCAAAGGAGT |
| *sub-1* qF | ACGGCATAGGAAAGCATTCG |
| *sub-1* qR | CAGGCATTGCAAAGAGTTCTC |
| *c-box* qchipF | CAATTTTGCAGCGTCATCGGTCT |
| *c-box* qchipR | TCAAGTCAAGCTCGTACCCACATC |
