# Supplementary material for: A HAD family phosphatase CSP-6 regulates the circadian output pathway in Neurospora crassa
Source: PLoS Genet. 2018 Jan 19;14(1):e1007192. doi: 10.1371/journal.pgen.1007192 (PMC5800702; doi:10.1371/journal.pgen.1007192)
Supplement: S2 Table — (DOCX) [file pgen.1007192.s012.docx]

**S2 Table**: **List of CSP-6 interactome identified by MS/MS from sliced gels of purified CSP-6**

| **Band #** | **Gene name ^a^** | **Description** | **Tp^b^** | **Up^c^** |
| --- | --- | --- | --- | --- |
| 1 | NCU08380T0 | plasma membrane phosphatase required for sodium stress response (449 aa) | 77 | 26 |
|  | NCU08380T2 | plasma membrane phosphatase required for sodium stress response (397 aa) | 58 | 29 |
| 2 | NCU08380T0 | plasma membrane phosphatase required for sodium stress response (449 aa) | 73 | 27 |
|  | NCU08380T2 | plasma membrane phosphatase required for sodium stress response (397 aa) | 66 | 23 |
| 3 | NCU08380T0 | plasma membrane phosphatase required for sodium stress response (449 aa) | 95 | 40 |
|  | NCU08380T2 | plasma membrane phosphatase required for sodium stress response (397 aa) | 40 | 35 |
| 4 | NCU08380T0 | plasma membrane phosphatase required for sodium stress response (449 aa) | 67 | 32 |
|  | NCU08380T2 | plasma membrane phosphatase required for sodium stress response (397 aa) | 63 | 28 |
| 5 | NCU10518T0 | general stress response protein Whi2 (298 aa) | 79 | 26 |
|  | NCU00489T0 | cytoplasmic ribosomal protein-10 (263 aa) | 15 | 14 |

a: Only list top two hit genes of each band

b: Tp: Total peptides

c: Up: Unique peptides
